# Material Safety of Styrene-*Block*-Ethylene/Butylene-*Block*-Styrene Copolymers Used for Cardiac Valves: 6-Month *In Vivo* Results from a Juvenile Sheep Model

**DOI:** 10.1093/ejcts/ezaf266

**Published:** 2025-08-01

**Authors:** Raimondo Ascione, Joanna R Stasiak, Daniel Baz-Lopez, Marta Serrani, Geoff D Moggridge

**Affiliations:** Bristol Medical School and Translational Biomedical Research Centre, Faculty of Health and Life Science, University of Bristol, Bristol BS2 8HW, United Kingdom; Department of Chemical Engineering and Biotechnology, University of Cambridge, Cambridge CB3 0AS, United Kingdom; Bristol Medical School and Translational Biomedical Research Centre, Faculty of Health and Life Science, University of Bristol, Bristol BS2 8HW, United Kingdom; Department of Chemical Engineering and Biotechnology, University of Cambridge, Cambridge CB3 0AS, United Kingdom; Department of Engineering, Durham University, DH1 3LE, Durham, UK; Department of Chemical Engineering and Biotechnology, University of Cambridge, Cambridge CB3 0AS, United Kingdom

**Keywords:** SEBS co-polymers, material safety, cardiac valves, preclinical testing, calcification

## Abstract

**Objectives:**

To assess the *in vivo* 6-month safety of styrene-*block*-ethylene/butylene-*block*-styrene (SEBS) block copolymers material used to make cardiac valves.

**Methods:**

Research-grade mitral valve prototypes made from SEBS29/SEBS20 copolymers (*n* = 7; 3 with heparin-coating) were implanted in juvenile sheep under cardiopulmonary bypass and kept for 6 months. No vitamin K antagonists were used. Anticoagulation included enoxaparin 1 mg/kg SC twice/day from day 1 until day 120 along with clopidogrel 300 mg once/day with food from day 1 until sacrifice. Safety measures included SEBS-related calcification, degradation, haemolysis, cytotoxicity, clinical pathology (biochemistry, complete blood count, coagulation), structural integrity, damage to surrounding tissue, overall animal health, and device embolization and function.

**Results:**

Surgery was feasible in all cases. Four animals reached the final 180 ± 5 days timepoint, while 1 needed non-SEBS related sacrifice on day 2, 1 suffered non-SEBS related death on day 81, and 1 needed sacrifice on day 169 due to prototype dysfunction. High-resolution X-ray, spectroscopy and histology showed absence of SEBS calcification, while gel permeation chromatography confirmed no SEBS degradation at 6 months. At histology, there was no SEBS-related calcification, thrombosis, cytotoxic or neoplastic degeneration, and no damage of the cardiac and downwards organs. Blood testing showed no haemolysis, while clinical pathology and animal health remained within normal reference intervals. The function of the research-grade mitral prototypes was clinically acceptable. The use of heparin-coating did not add benefit.

**Conclusions:**

This preclinical *in vivo* study in juvenile sheep confirms the 6-month safety of SEBS29/SEBS20 material used to make cardiac valves. A future early feasibility study is warranted to confirm long-term durability, haemocompatibility, and function in humans.

## Graphical abstract

**Figure:**
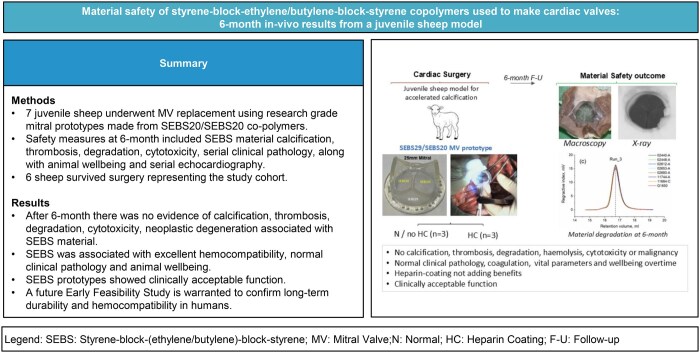

## INTRODUCTION

Currently 1.3 million patients require cardiac valve replacement globally every year^1–5^ with 4-5 times increase predicted by 2050 due to elderly.^3^^,^^6^^,^^7^ Concomitantly, rheumatic disease affects 35 million patients in undeveloped countries.^4^ Current mechanical valves have excellent durability but require life-long anticoagulation with vitamin K antagonists leading to 3%-4% thrombo-embolic/haemorrhagic events/year^7^ and teratogenicity during pregnancy. Biological valves are biocompatible^1^^,^^5–9^ but are affected by early calcification end leaflet degeneration leading to poor durability, readmissions, and reoperations.^5–10^ Hence, new materials and technologies are being tested to develop novel heart valves that might combine good biocompatibility with excellent durability, possibly through simplified manufacturing and quality control.

Styrene-block-(ethylene/butylene)-block-styrene (SEBS) block copolymer are stable synthetic thermoplastic elastomers amenable to high-throughput injection moulding as previously reported by our group and others.^11–16^ SEBS contain polystyrene hard domains forming physical cross-links in a continuous elastomeric phase, therefore providing strength, along with flexibility and elasticity. SEBS with a 20%-33% styrene content self-assembles into a cylindrical microstructure which trigger structural anisotropy with different mechanical properties in 2 orthogonal directions via hot melt processing,^16^ a feature resembling the structure of human heart valves.^17^ So far, applications of SEBS in medicine have included only transdermal patches.^18^ We have developed a research-grade aortic heart valve made from SEBS29 and SEBS20. Strips of SEBS material tested *in vitro* in Badimon chambers with human blood confirmed very good haemocompatibility.^11^ The latest J6 aortic valve prototype demonstrated a long-term durability of 1.2 billion cycles at the *in vitro* pulse duplicator bench testing, equivalent to 35 years of durability *in vivo*, with a functional performance comparable with the best-in-class biological valves at bench testing along with confirmation of short-term *in vivo* feasibility, haemocompatibility, and function.^11^ The next step towards regulatory submission was the assessment of SEBS safety in a preclinical *in vivo* study. Hence, the aim of the present 6-month preclinical *in vivo* study was to assesses the safety of SEBS materials when used to make cardiac valves in a juvenile sheep model known to trigger accelerated calcification.^19^

## METHODS

### Ethical statement

The study was undertaken at the accredited preclinical testing facility Veranex (previously known as IMMR) (Paris, France) in conformity with ISO 10993-2, the European Convention for the Protection of Vertebrate Animals Used for Experimental and Other Scientific Purposes, Council of Europe ETS 123, the French legislation, and the Guide for the Care and Use of Laboratory Animals, NRC 2011. In addition, the experiments were designed in compliance with ISO 5840-1:2021(en)—Cardiac valve prostheses.

Twenty-four SEBS29/SEBS20 mitral valve (MV) prototypes (Test Article) were manufactured in our research lab (all 25 mm size; **Figure S1A**; molecular characteristics in **Table S1**), adapting the previous J6 aortic valve design^11^ to add a SEBS29 skirt/sewing ring with pre-made holes with no fabrics. The rationale for not using fabrics was based on the manufacturing technology allowing to add polymeric sawing skirt within the same single molding process, along with the objective of minimizing the risk of fabrics triggering thrombosis or housing of bacteria from the bloodstream. Twelve of the 24 Test Articles were kept for bench-testing aiming for 100 million cycles, while the remaining 12 were used for this material safety study. Of these, 6/12 were heparin-coated (HC 1-6), while the remaining 6 were normal (N1-6). All 12 MVs prototypes underwent clinical-grade dry packaging and ethylene oxide (EO) sterilization. The goal was to achieve 6 animals (*n* = 3HC and *n* = 3N) surviving surgery to generate *in vivo* material safety data over a 6-month period using a predefined lists of assessment methods and acceptance criteria in line with regulatory expectations.

### Anaesthesia, surgery, and postoperative complications

Coded juvenile sheep (Ile de France, mixed male/female; 5-11 months old; weight range 38-52 kg) were subjected to general anaesthesia (GA) and surgery as reported in **Supplemental File** and **Table S2**. Surgery was via left thoracotomy under heparinization, and cardiopulmonary bypass (CPB) established via left carotid artery and jugular vein cannulation. A supra-annular implant of all SEBS mitral prototypes was carried out through the left atrial auricle on the vented beating heart (**Figure S1B and C**). Postoperative care was according to standard operating procedures (SOPs). Pain control and antibiotic treatment are reported in **Supplemental File**. No vitamin K antagonists were used. Enoxaparin 1 mg/kg SC twice/day was used from day 1 until day 120 and clopidogrel 300 mg/day with food from day 1 until sacrifice. In case of severe debility, animals were sacrificed for humane reasons according to predefined SOP if unable to feed and/or ambulate or if in class IV NYHA heart failure despite adequate treatment (early sacrifice). Any death occurring before the scheduled 180 ± 5 days was classified as early death. Non-material/SEBS related sacrifice or death was defined as an event not related to the SEBS material.

### Assessment of study safety objectives

The study safety objectives included: calcification, degradation, haemolysis, cytotoxicity, clinical pathology, structural integrity, damage to surrounding tissue/structure, overall animal health, and device embolization or migration, or fragmentation and function. For each safety objective assessment timepoint, assessment methods and acceptance criteria are shown in **Table 1**.

**Table 1.** Assessment of Study Safety Objectives

Safety test/endpoint	Assessment timepoint	Assessment method	Acceptance criteria
Leaflet calcification	Post-explant	Macroscopic assessment High-resolution X-ray (Faxitron) Spectroscopy (calcium and phosphorus level) Histopathology assessment	No clinically unacceptable Test Article leaflet calcification
Material degradation	Post-explant	Gel permeation chromatography (GPC)	No clinically unacceptable material degradation
Haemolysis	Day 0 Day 14 ± 2 Day 30 ± 2 Day 120 ± 5 Day 180 ± 5	Blood sampling for analysis of Plasma-free haemoglobin	No clinically unacceptable haemolysis
Device cytotoxicity: Foreign body response (pannus formation, tissue overgrowth, malignant cells)	Post-explant	Macroscopic assessment Histopathology assessment	No clinically unacceptable foreign body response to the Test Article
Clinical pathology Complete blood count Biochemistry Coagulation profile	Day 0 Day 14 ± 2 Day 30 ± 2 Day 120 ± 5 Day 180 ± 5	**Complete blood count**: RBC, haemoglobin, haematocrit, MCV, MCHC, MCH, white blood cells (WBC), WBC differential counts (neutrophils, lymphocytes, monocytes, basophils and eosinophils), platelets, and reticulocytes **Biochemistry**: glucose, creatinine, urea, calcium, chloride, sodium, potassium, bicarbonate, phosphorus, plasma proteins, magnesium, total bilirubin, GGT, AST, ALT and alkaline phosphatase **Coagulation profile**: fibrinogen, prothrombin time (PT) and activated partial thromboplastin time (aPTT)	No clinically unacceptable derangement of CBC, biochemistry, coagulation profile vs normal reference intervals
Structural integrity	Post-explant	Macroscopic assessment High-resolution X-ray (Faxitron) Histopathology assessment	No clinically unacceptable Test Article structural damage
Damage to the surrounding tissues/structure	Post-explant	Macroscopic assessment Histopathology assessment (including for calcification (von Fossa staining) and neoplastic growth (H&E)	No clinically unacceptable Test Article-related damage to the surrounding and adjacent anatomical structure
Overall animal health	Over 6-month and post-explant	Clinical observations Body weight and temperature Clinical pathology Gross necroscopy Macroscopic assessment Histopathology assessment	No clinically significant Test Article-related adverse events
Device migration/fragmentation Device function	Day 0 Day 14 ± 2 Day 30 ± 2 Day 120 ± 5 Day 180 ± 5 Post-explant	Echocardiography Macroscopic assessment Histopathology assessment	No clinically unacceptable Test Article embolization or migration or function

Abbreviations: ALT: alanine aminotransferase; aPTT: activated partial thromboplastin time; AST: aspartate aminotransferase; GGT: gamma glutamyl transferases; H&E: haematoxylin and eosin; MCH: mean corpuscular haemoglobin; MCHC: mean corpuscular haemoglobin concentration; MCV: mean corpuscular volume; PT: prothrombin time; RBC: red blood cells; WBC: white blood cells.

### Necroscopy, macroscopy, and histology

The heart inclusive of the valve and all targeted organs were sampled, imaged, assessed macroscopically and histologically, and then reported by expert independent pathologists at the accredited preclinical facility based on their SOPs and their independent clinical assessment.

The hearts were excised and downwards organs sampled (lungs, adrenal glands, liver, spleen, brain, kidney, plus any other abnormal finding) (**Supplemental File**). The SEBS29/SEBS20 valves were explanted embedded within surrounding tissue and macroscopically assessed for calcification, thrombosis, infection/abscess/vegetation, cusp haematoma, pannus, cusp retraction, and tears/perforations. High-quality photographs were taken from atrial (inflow) and ventricular (outflow) sides. Macroscopic outcomes were reported as absent or present. After fixation in 4% formaldehyde, samples were embedded in paraffin. Three µm sections were stained with Haematoxylin-Eosin and Saffron (HE&S). The 3 cusps of each valve (SEBS20), identified as lateral (L), cranial (C), and medial (M), were excised. A longitudinal strip from the middle of each cusp was used for histology including paraffin sections, HE&S, and special stains (ie, Movat Pentachrome, Masson Trichrome for connective tissue, Alizarin Red and for calcium, and Azan Mallory for fibrin). The remaining parts of each cusp were assessed for calcium and phosphorus content. All sections were digitalized at x20 objective using Nanozoomer (Hamamatsu, Japan). Microscopic outcomes were qualitatively scored as absent or present; if changes were present, they were graded focal, multifocal, locally extensive or diffuse using a 1 to 5 grade severity scale (ie, minimal, mild, moderate, marked, or severe). Additional H&E and von Kossa staining was undertaken on cardiac tissue sections either directly interfacing with SEBS29 or 0.5 cm away from it (control) to assess for calcification and cell proliferation.

### SEBS29/SEBS20 material related safety objectives

#### Calcification

High-resolution radiography images (*Faxitron*) were taken and calcification scored as absent or present. Calcium and phosphorus levels were measured by atomic absorption spectroscopy (AAS)^19^ using an ICP AES iCAP 6500 Duo system (Thermo Scientific) on pooled samples from each SEBS20 l, C, and M cusp from each valve and expressed as micrograms per milligram (%m) dry weight. Macroscopy and histology assessment were as reported above.

#### Degradation

Gel permeation chromatography/size exclusion chromatography (GPC/SEC) was used on a Viscotek GPCmax (Malvern Panalytical), equipped with 270 dual detector system, Viscotek UV detector 2500, and RI detector VE 3580. About 10 mg of SEBS29 polymer was cut from each prototype implanted (*n* = 7) and from a raw SEBS29 sample and dissolved in tetrahydrofuran (THF) GPC grade (Fisher Scientific) to form an eluent of 1 mg/mL concentration. A defined volume of the sample solution was filtered and loaded onto the column using an autosampler. The flow rate was kept at 1 mL min^−1^. The separation column was LT5000L, Mixed, Medium Org, packed with microparticulate material (styrene-divinylbenzene gel) of 10 µm particle size (**Supplemental File**).

#### Damage to surrounding tissue/structure and downwards organs

Von Kossa staining was used to assess for calcification of surrounding myocardial sample, which were incubated in 1% silver nitrate, ultraviolet light applied for 20 min, followed by washing with 5% sodium thiosulfate for 5 min, and counterstained with nuclear fast red for 5 min. Magnification X40 scans were obtained by M8 Microscope and Scanner. Positive controls included millimetric sections of human saphenous vein in high-glucose DMEM supplemented with 10% foetal bovine serum, 100 U/mL penicillin/streptomycin, and 2.5 mM L-glutamine in the presence of 10 mM β-glycerophosphate, 8 mM CaCl2 and 50 µM ascorbic acid for 7 days.^20^ Calcium was identified by the presence of black stained precipitate. H&E staining was used to assess cytotoxicity and cell proliferation.^21^ Myocardial specimens were divided into 3 regions of interest (ROIs): the region interfacing SEBS29 and 2 adjacent regions 2 mm away not directly interfacing SEBS29. The nuclei number was determined at X20 magnification pictures obtained by M8 Microscope and Scanner. ImageJ software was used for manual nuclei quantification.

#### Clinical pathology

Serial blood collection occurred before surgery, 14, 30, 120, and 180 days after surgery and included the following tests:

*Complete blood count (CBC):* red blood cells (RBC), haemoglobin, haematocrit, mean corpuscular volume (MCV), mean corpuscular haemoglobin concentration (MCHC), mean corpuscular haemoglobin (MCH), white blood cells (WBC), WBC differential counts (neutrophils, lymphocytes, monocytes, basophils and eosinophils), platelets, and reticulocytes.

*Biochemistry:* glucose, creatinine, urea, calcium, chloride, sodium, potassium, bicarbonate, phosphorus, plasma proteins, magnesium, total bilirubin, gamma glutamyl transferases, aspartate aminotransferase (AST), alanine aminotransferase (ALT), and alkaline phosphatase.

*Coagulation screen:* prothrombin time ratio (PR) fibrinogen and activated partial thromboplastin time (aPTT).

#### Haemolysis

This objective involved blood sampling for analysis of plasma-free haemoglobin.

#### Structural integrity

This objective was based on macroscopy, high-resolution X-ray, and histology.

#### Overall animal health

This objective was assessed via clinical observations, body weight and temperature, clinical pathology, gross necroscopy, macroscopy, and histology assessments.

#### Device embolization, migration, or fragmentation and device function

This objective was assessed via echocardiography, macroscopy, and histology.

### Statistical analysis

As this was a material safety study, no control group was used. Safety measures related to clinical pathology were evaluated vs normal reference intervals. All other safety measures were assessed against predefined acceptance criteria (**Table 1**). The sample size of 6 animals and lack of a control group was based on preliminary discussions with the regulator. Reported findings are mainly descriptive and presented as means ± standard deviation or as percentage or proportions.

## RESULTS

Seven animals were implanted with 7 SEBS research-grade polymeric heart valve in mitral position. Of these, 3 were heparin-coated valves (study animals 02653, 11744, 02612) and 4 were normal (study animals 02440, 02448, 02680, 11684). At surgery, the implantation procedures were smooth. No clinically significant Test Article-related adverse events occurred during the implantation procedures, and all Test Articles were implanted as planned in the intended position and location. Three of 7 mismatching occurred (study animals 02448, 02612, 02440) due to small MV annulus. Study animals 02680 (N), 11744 (HC), 02612 (HC), and 02653 (HC) completed their 6-month follow-up and were respectively sacrificed at day 180, 181, 176, and 180. Study animal 2440 (N), 1 of the 3 animals with intraoperative mismatching, was electively sacrificed due to respiratory distress at day 169 due to SEBS-related valve dysfunction. Study animal 02448 (N), also 1 of the 3 animals with intraoperative mismatching, died of cardiac arrest during the intermediate control anaesthesia at day 81. Macroscopy showed an issue occurred at surgery with 1 of the sutures used to stitch the valve looping around/blocking 1 of the valve posts with associated small tears (**Figure S2**). Study animal 11684 (N) was electively sacrificed at day 2 due to non-SEBS related diminished mentation, abnormal neurological signs, and seizures. At macroscopy, the valve 11684 was intact. Hence, the 6 animals surviving surgery represented the study cohort for this 6-month material safety study. However, samples from prototype 11684 were included in the material degradation test.

### Macroscopy/necroscopy

The SEBS prototypes, heart, lungs, adrenal glands, liver, spleen, and brain were sampled and put in 4% neutral buffered formalin for all study animals, except study animal 11684 in which no samples were taken and study animal 02448 in which no heart samples were taken. Additional macroscopic findings were sampled in study animals 02612 (right kidney), 02440 (right kidney), and 02680 (left kidney and liver).

### SEBS29 and SEBS20 materials

**Figure 1.**
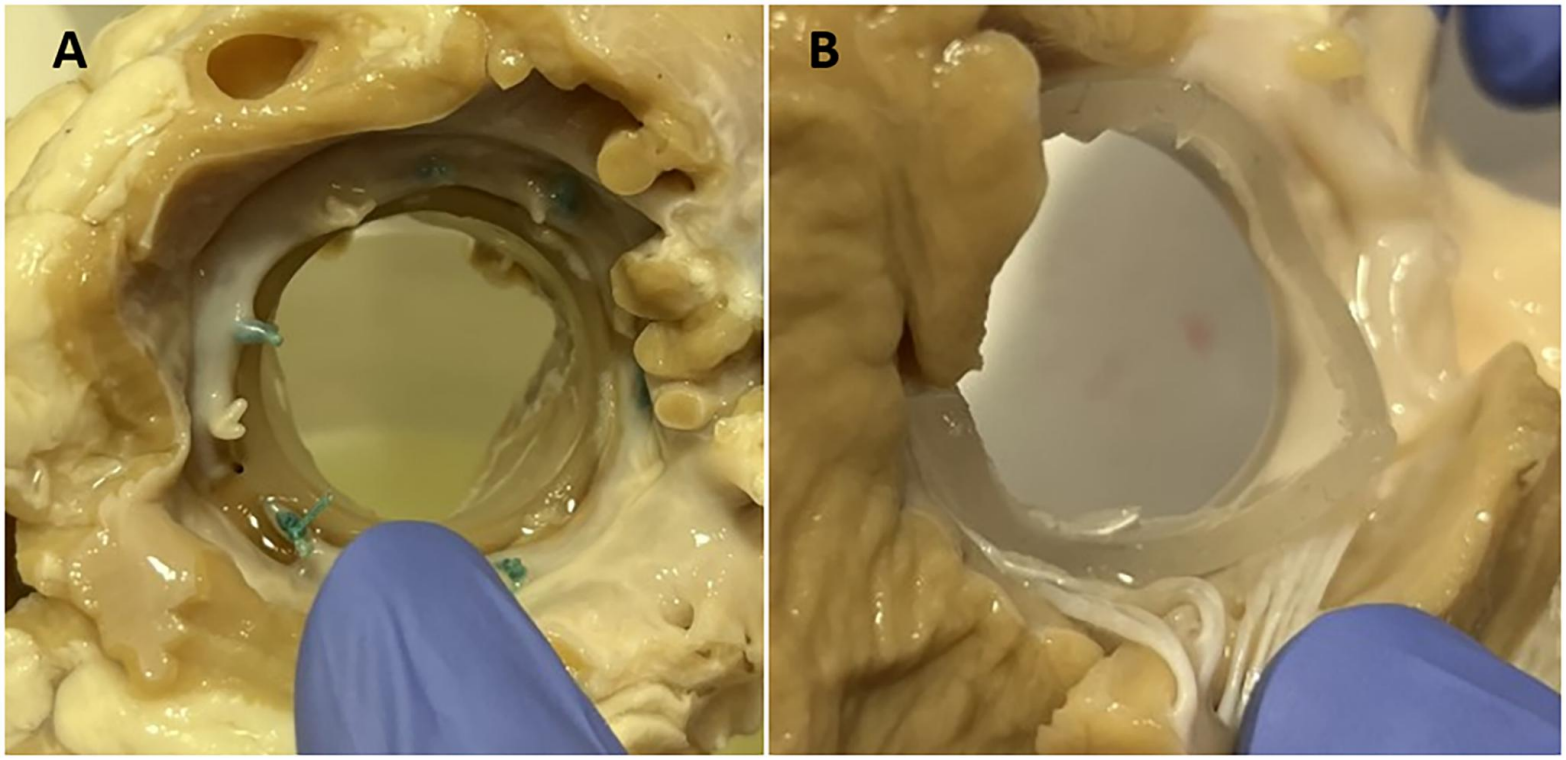
Macroscopy of SEBS29: Representative Image of 11704 Prototype. Inflow (A) and outflow (B) views showing no calcification, thrombosis, pannus, or structural abnormalities of the SEBS29 material or the interfaced cardiac tissue

**Figure 2.**
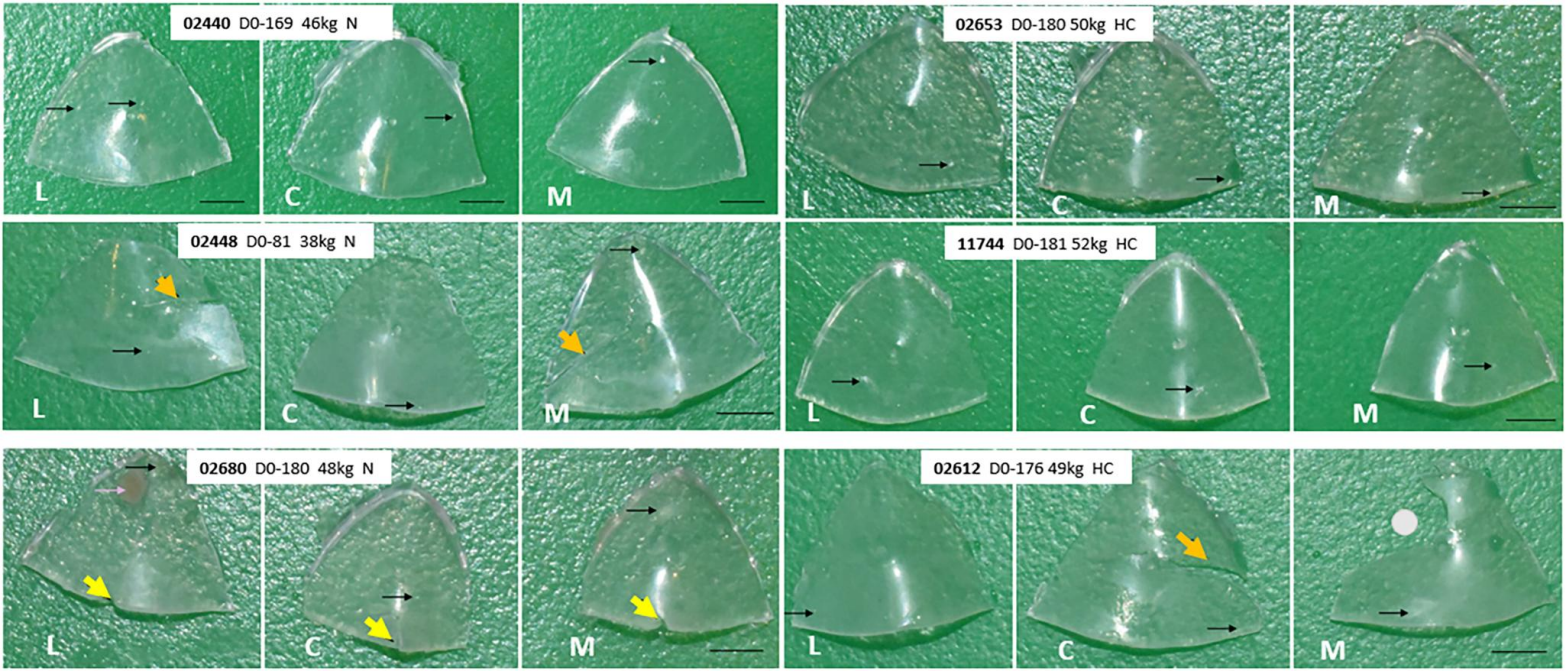
Macroscopy of SEBS20. Inflow views of all leaflets; black arrows show tiny trace of pannus; purple arrow shows a small fibrin deposit on 1 leaflet; yellow arrows show small tears; orange arrow shows large tears; grey dot shows a missing part in 1 leaflet. Scale bar: 0.5 cm. L: Lateral cusp; C: Cranial cusp; M: Medial cusp. Legends report study animal, termination day, weight at surgery, N (normal) or HC (heparin-coated) sub-groups

Macroscopy of SEBS29 is shown in **Figure 1** and **Figure S3**, while macroscopy of SEBS20 is shown in **Figure 2** and **Figure S3**. None of the 6 SEBS29 rings/skirts or the 18 SEBS20 leaflets showed obvious calcification, thrombosis, pannus, cusp retraction or perforation. All valves appeared firmly anchored to the MV annulus. One small fibrin deposit (3 × 3 mm) and tiny leaflet tears at the free margins were observed on prototype 02680. Small or moderate leaflet tears at the interface SEBS29/SEBS20 were observed in prototypes 02448 and 02612, along with a missing leaflet part for 02612 (**Figure 2**).

### Material safety measures

#### SEBS29/SEBS20 calcification

**Figure 3.**
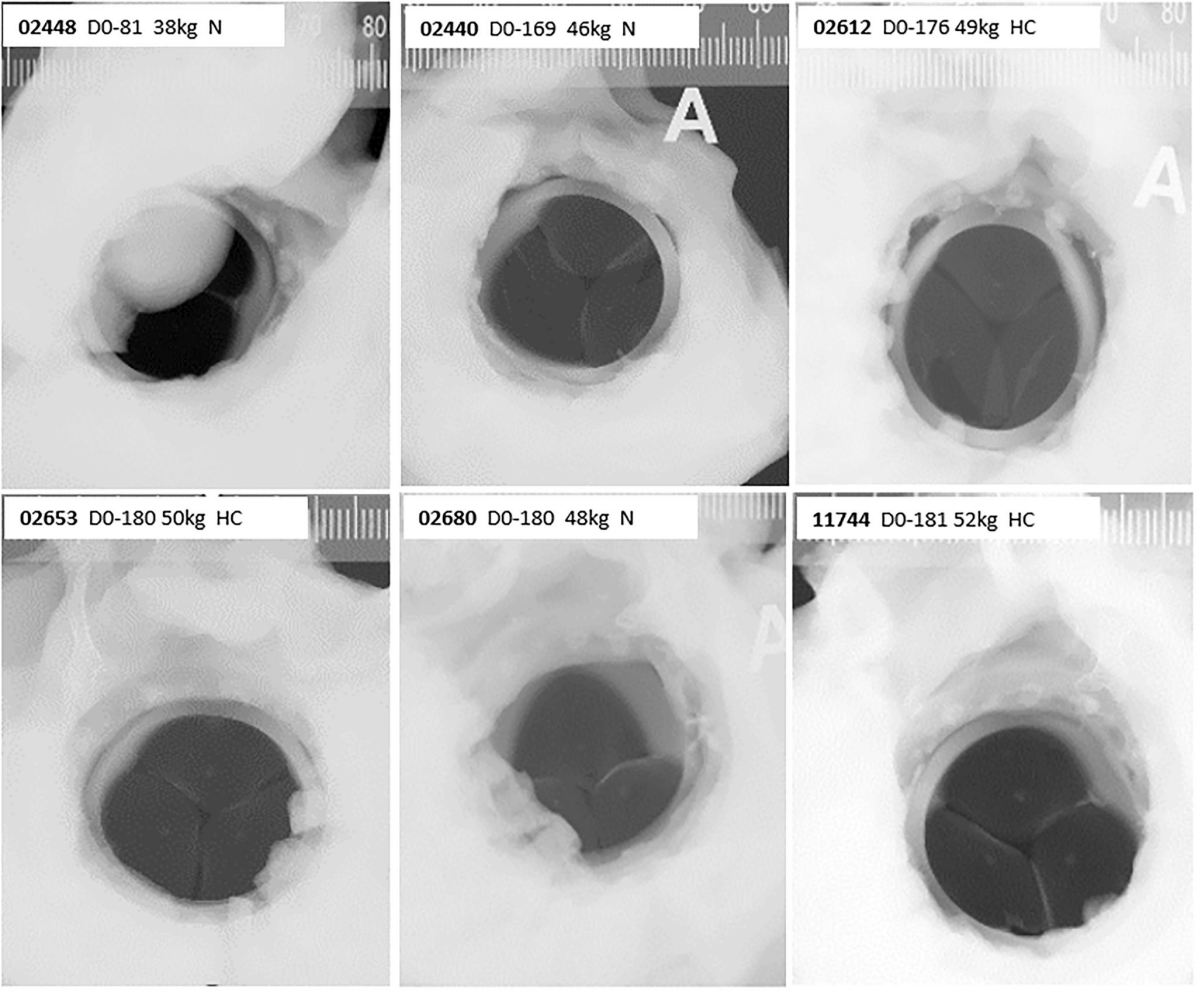
High-resolution X-ray (Faxitron) Images. No calcification seen on any SEBS29/SEBS20 parts. Legends report study animal, termination day, weight at surgery, N (normal) or HC (heparin-coated) sub-groups

High-resolution X-ray showed absence of calcification at 6 months for all 18 cusps (SEBS20) and 6 skirts/rings (SEBS29) (**Figure 3**). Mean dry weight was 121.72 ± 9.9 mg. Mean calcium content was 0.021 ± 0.004% of dry weight. Mean phosphorus content was not traceable (>0.100 ± NA %m). There was no difference in calcium and phosphorus contents between prototypes explanted on day 81 (study animal 02448), day 169 (study animal 02440), or days 180-181 (study animals 02680, 11744, 02612, and 02653) (**Table 2**).

**Table 2.** Calcium and Phosphorus Levels

Study animal	Timing	Subgroup	Total dry weight (mg)	Ca (%m)	P (%m)
02440	Early death (day 169)	N	118.965	0.022	<0.100
02448	Non-material related Early death (day 81)	N	125.835	0.019	<0.100
02680	Completion of follow-up (day 180)	N	103.706	0.027	<0.100
11744	Completion of follow-up (day 181)	HC	129.125	0.023	<0.100
02612	Completion of follow-up (day 176)	HC	110.396	0.023	<0.100
02653	Completion of follow-up (day 180)	HC	125.650	0.016	<0.100
Mean			121.724	0.021	<0.100
Standard deviation			9.963	0.004	N.A.

Abbreviations: Ca: Calcium; P: Phosphorus; %/m: percentage of dry weight.

#### SEBS29 degradation

**Figure 4.**
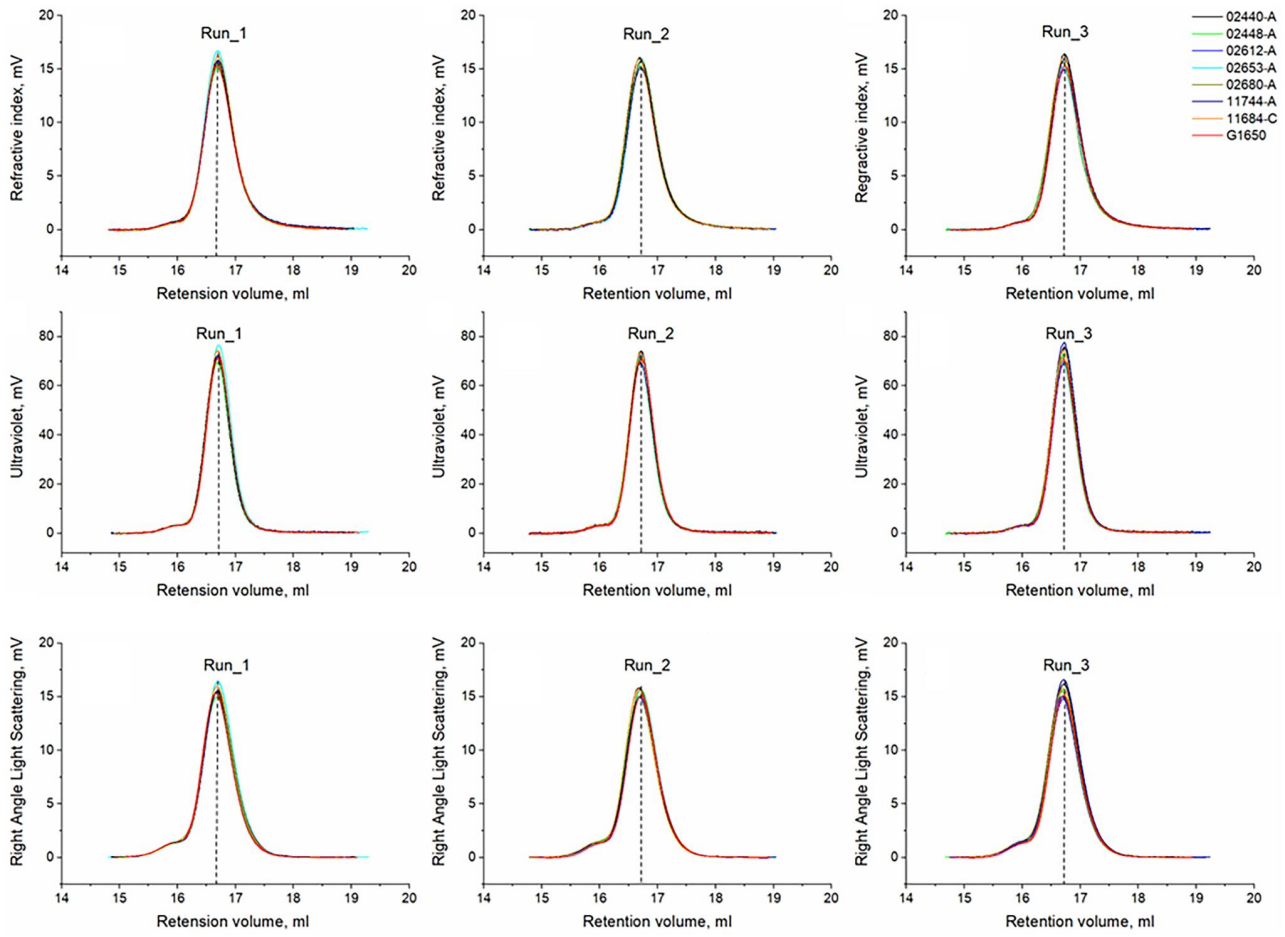
SEBS29 Degradation. Triple detector chromatograms of SEBS29 samples from all explanted valves and the non-implanted raw material (G1650)

There was no SEBS29 material degradation across the 7 prototypes explanted from day 2 to day 181 after surgery and no difference was observed vs the non-implanted raw material. Hence, all the SEBS29 samples assessed generated identical GPC elution curves (**Figure 4**). The observed trivial variations between the Mw and Mn values were below ±5% (**Table S4**) and within the accepted 3%-15% error for this measurement.^22^

#### Cardiac tissue interfacing SEBS29 and downwards organs

There was no calcification of the myocardium interfacing SEBS29 or the non-directly interfacing regions (**Figure S4**). There was no difference between groups in nuclei count for cytotoxicity or cell proliferation and tissue structure indicating that direct long-term interfacing with SEBS29 was not associated with cytotoxicity or abnormal cell proliferation (**Figure S5**, **Table S5**). All downwards organs tested showed no evidence of calcification, thrombosis, fibrosis, cytotoxicity, or neoplastic formation based on negative staining for Alizarin Red (calcium), Movat Pentachrome/Masson Trichrome (collagen/fibrosis), and H&E (cytotoxicity/cell proliferation). Minor and non-specific changes observed are shown in **Figure S6**.

#### Clinical pathology

All measurements overtime were within normal reference intervals, with very few being slightly below (**Table S6**).

#### Biochemistry

Biochemistry values remained within normal reference intervals indicating that using SEBS29/SEBS20 was non associated with renal or liver injury while glucose metabolism and electrolytes homeostasis remained normal.

#### Haemolysis

Plasma-free haemoglobin levels remained normal overtime, indicating absence of haemolysis/red cell injury.

#### Complete blood count

CBC values remained within normal reference intervals indicating absence of anaemia, neutropenia, infective/sepsis status, or blood/bone marrow disorders/malignancies.

#### Coagulation and heparin coating

Levels of PR, fibrinogen, and aPTT remained within normal reference intervals and rarely slightly below, indicating absence of clinically unacceptable derangement of coagulation overtime. This also confirmed absence of actively induced anticoagulation. Heparin-coating did not affect these measurements.

### Overall animal health

All animals remained in good well-being conditions, with normal appetite and weight gain over time (**Figure S7**) along with normal serial clinical pathology (**Table S6**). Vital parameters remained within normal reference intervals (data not shown but available). A part the expected trivial weight loss for few days after surgery, there was 20% average weight gain (approx. 11 kg) vs baseline (**Figure S7**).

### Echocardiography

Echocardiography was acquired at all timepoints and is reported for the 5 animals not suffering premature non-SEBS related death (**Table 3**). Transvalvular mean and peak gradients were clinically acceptable and better than those expected by ISO standards for preclinical studies. Transvalvular regurgitation was mild across most of the timepoints, while no periprosthetic leaks were observed. In 3/5 study animals, the prototypes functioned very well up to the final 180 ± 5 days timepoint. Two study animals affected by moderate mismatch at surgery developed moderate regurgitation at day 120 and subsequently cusp tears at day 169 (study animal 02440) and day 176 (study animal 02612).

**Table 3.** Function of 25 mm SEBS Mitral Valve Prototype at 6 Months Follow-up in Sheep

Outcome measures (25 mm SEBS prototype)	Day 14 ± 2 (*n* = 5)	Day 30 ± 2 (*n* = 5)	Day 120 ± 2 (*n* = 5)	Day 180 ± 5 (*n* = 4)
MG of all 5 valves (mmHg; mean ± SD)	6.0 ± 2.8	5.3 ± 2.2	8.2 ± 3.0	12.6 ± 4.4
PG of all 5 valves (mmHg; mean ± SD)	9.5 ± 3.3	9.5 ± 2.1	12.0 ± 4.2	20.3 ± 9.9
MG valves with no mismatch (mmHg; mean ± SD) (*n* = 4)	5.3 ± 1.7	5.0 ± 2.2	7.3 ± 2.5	10.9 ± 2.5
PG valves with no mismatch (mmHg; mean ± SD) (*n* = 4)	8.5 ± 2.4	9.0 ± 2.4	10.8 ± 3.7	16.3 ± 2.6
Grade of regurgitation (*n* = 5):				
Mild	5	5	4	3
Moderate	0	0	1	0
Moderate/Severe	0	0	0	1^a^

a One of the 5 study cases was culled at day 169 due to haemodynamic instability with echocardiography showing severe regurgitation, making the number of cases with moderate/severe MR 2/5 for the whole study.

Abbreviations: MG: mean transvalvular gradient; PG: peak transvalvular gradient; SD: standard deviation.

## DISCUSSION

This study confirms the safety of SEBS29/SEBS20 materials when used to make cardiac valves. The study was conducted at an accredited testing facility according to the Guide for Care and Use of Laboratory Animals,^23^ ISO 10993-1:2020,^24^ ISO 10993-2:2006,^25^ ISO 10993-4:2017,^26^ ISO 10993-6:2016,^27^ and ISO 10993-11:2017.^28^

There was no calcification at 6 months based on macroscopy, high-resolution X-ray, and calcium/phosphorus content. While this may suggest that future cardiac valves made from SEBS29/SEBS20 may not be affected by early calcification, a limitation of current surgical and percutaneous biological valves,^8^^,^^9^ this suggestion require validation in future clinical studies in humans. Of note, 4-6 months of *in vivo* preclinical testing represents the period expected by the regulator when a new material is used, possibly using a juvenile sheep model regarded as the best preclinical model of accelerated calcification.^19^ Accordingly, the same *in vivo* model was used when assessing *in vivo* calcification in sheep over 8-month between clinical-grade Perimount biological MV vs a new configuration with a novel anti-calcification treatment made by Edwards Life Science.^19^ High-resolution X-ray showed calcification in 57% of the cusps and 50% of the commissures of the Perimount group (*n* = 14) vs 24% of the cusps and 35% of the commissures in the anti-calcification group (*n* = 17). Calcium content, measured via spectroscopy, was 0.68% and 0.19% of dry weight in the Perimount vs the anti-calcification group respectively. The present study testing SEBS29/SEBS20 research-grade prototypes in the same sheep model showed no calcification at the high-resolution X-ray. In addition, the calcium content at 6 months appears to be 34 and 9.5 times lower than the amount reported in the Perimonut and the anti-calcification group respectively in the paper by Flameng *et al.*^19^

Another finding was the absence of thrombosis related to SEBS29/SEBS20 materials, along with the absence of thromboembolic events such as stroke, limb ischaemia, acute liver/kidney failure, and negative macroscopy and histological evaluation of the hearts and downwards organs. Of note, these results were obtained despite no use of vitamin K antagonists. It might be argued that the absence of thrombosis and thromboembolism in this study was due to the use of enoxaparin and clopidogrel. We used a relatively low dose amount of enoxaparin at 1 mg/kg twice/day and clopidogrel 300 mg once/day with food, both without loading doses, for the first 4 months, followed by only clopidogrel 300 mg once/day with food for the last 2 months. To assess the anticoagulation effect of this dosage on the animals, we assessed serially their coagulation (PR, fibrinogen, and aPTT levels) over the 6-month period. The results suggest that all markers of coagulation remained mostly within reference intervals (**Tables S6**), indicating absence of therapeutic effect for the dosage of enoxaparin and clopidogrel used. Accordingly, a landmark prospective study by Weigand *et al.* on efficient anticoagulation and antiplatelet strategies in sheep^29^ confirms that the dosage of enoxaparin and clopidogrel used in the present study was too low and could not have triggered a tangible anticoagulation therapeutic effect. They reported that using enoxaparin in sheep at 1 mg/kg twice/day, along with a loading dose of 2 mg/kg, did not trigger a therapeutic effect and that it took a much higher dose at 3 mg/kg twice/day along with a loading dose to see a therapeutic effect.^29^ Regarding clopidogrel, they tested 3 increasing doses with food at 150 mg twice/day, 225 mg twice/day, and 375 mg twice/day, along with loading doses of 300 mg, 450 mg, or 600 mg respectively. The results showed that none of the 3 doses of clopidogrel triggered a tangible platelet inhibition when assessed by light transmission aggregometry, and that only in 2/5 sheep tested and receiving 325 mg twice/day plus 600 mg bolus there was a low level of PLT inhibition.^29^

Taken together, these results indicate very good *in vivo* haemocompatibility of the SEBS29/SEBS20 materials at 6 months after surgical implant in sheep, despite the use of low dose enoxaparin and clopidogrel and despite their serial coagulation profile remained within reference intervals over the 6-month period. Another relevant finding of this preclinical study is that adding HC in a subgroup (*n* = 3) was not associated with any additional benefit of the relevant measures of thrombosis, thromboembolism or coagulation profile vs the SEBS alone (*n* = 3).

Overall, the excellent preclinical haemocompatibility and lack of calcification of these SEBS prototypes observed in sheep over a 6-month period may represent an ideal scenario if similar findings were to be confirmed in humans in future clinical studies. Current mechanical valves are very durable but require mandatory anticoagulation with vitamin-K antagonists, with related risk of thrombo-haemorrhagic events.^30^^,^^31^ Biological valves have much better haemocompatibility, although suffer from early structural deterioration and calcification, with some ongoing debate on the best anticoagulation regimen to use^30^^,^^31^ given some recent evidence of subtle early thrombosis.^32^^,^^33^

All tested SEBS29 samples in this study generated nearly identical GPC elution curves indicating absence of SEBS degradation overtime. GPC/SEC technology is based on separation of molecules by their hydrodynamic radius or volume, that is, the molecules are separated by size, with the largest eluting first and the smallest last. The eluted molecules pass through a series of detectors and are automatically analysed by software to determine molecular weight, intrinsic viscosity, and molecular density. This test confirmed that all assessed SEBS29 samples showed the maximum of the elution profile at the same retention time. The average retention time for the maximum signal intensity was 16.7 min, indicating that the average molecular weight of all the assessed SEBS29 samples was the same and therefore that the explanted material had not chemically degraded over time and had not broken into shorter chains after 6 months *in vivo*.

Regarding biological safety and cytotoxicity, the SEBS29/SEBS20 materials did not trigger tangible pannus formation, cell proliferation, calcification, or damage of surrounding cardiac tissue or sampled organs. Minor pannus was seen only on the inflow side of the prototypes by the surgical sutures but not on the leaflets. This feature may suggest that SEBS29/SEBS20 are biologically inert materials, although this requires future confirmation.

Using SEBS29/SEBS20 materials did not derange clinical pathology over the 6-month period with biochemistry, complete blood count, haemolysis, and coagulation tests levels remaining within normal reference intervals. This normal clinical pathology was associated with evidence of good overall animal health, with normal vital parameters, nutrition, and weight gain over the 6-month period, along with absence of strokes, infections, anaemia, blood or bone marrow cancer, renal or liver failure.

The function of these SEBS29/SEBS20 research grade prototypes was in keeping with the expected ISO standards for new materials. No perivalvular regurgitation was reported, indicating good anchoring of the valve to the mitral annulus. Regarding transvalvular regurgitation, this was mild across the valves till day 120 timepoint, when 1 valve showed moderate regurgitation. Subsequently, at day 169, another valve showed severe regurgitation with leaflet tears leading to early sacrifice. Regarding gradients, there was a gradual increase over the 6-month period, which might be due to a variety of factors including excessive flexibility of these early prototypes, or their small 25 mm size, or their manufacturing in a research lab, that is, not being a finite clinical grade product, or the presence of some mismatch at surgery. These aspects have triggered refinements in design towards the clinical grade device and a future submission to the regulator.

This study has several limitations. The sample size was small, although in line with expectations by the regulator for material safety studies. Safety data were derived from all 6 animals surviving surgery using regulatory methods of assessment. A control group is not required for this type of study as the focus is to assess the safety of the new material using methods of assessment and acceptance criteria predefined by the regulator. A possible limitation of this study is the use of the sheep model. However, using juvenile sheep is regarded by the regulator and tier 1 companies as the most effective preclinical model of *in vivo* accelerated calcification.^19^ It might also be argued that 180 days of follow-up *in vivo* is not a long enough period for material safety evaluations. However, under ISO 10993 and other regulatory standards, 180-day animal studies can be used to support long-term material safety if making a case for future regulatory approval. Yet, it is important to appreciate that the long-term findings of this material safety study are not synonymous of long-term safety in humans, for which future clinical trials are warranted. Another limitation was the availability of only a 25 mm size for the prototypes. Also, the 2 male juvenile (5 months old) sheep implanted at the start of the study weighed less than 50 kg, resulting in a too small mitral annulus for the Test Article implantation. These aspects might have contributed to some mismatching observed at surgery in 3/7 cases. A final limitation might be the selected *in vivo* test system. There are anatomical and physiological differences between the healthy ovine model and the targeted diseased human patient that could influence the implantation and performance of any Test Article including the heart chambers in sheep being smaller than that expected in the relevant human patient population, the mitral leaflets are longer and thicker in humans than in sheep, and the ovine model is known for the accelerated calcification process.

In conclusion, this preclinical *in vivo* study in juvenile sheep confirms the 6-month safety of SEBS29/SEBS20 material when used to make cardiac valves. An early feasibility study is warranted to confirm long-term durability, haemocompatibility, and function in humans.

## Supplementary Material

ezaf266_Supplementary_Data

## Data Availability

The raw material safety data are in the Final Report released by the testing facility, which can be made available following reasonable request. Material degradation raw data undertaken in Cambridge and cardiac tissue histology undertaken in Bristol can be also made available following a reasonable request.
