# Supplementary material for: Material Safety of Styrene-Block-Ethylene/Butylene-Block-Styrene Copolymers Used for Cardiac Valves: 6-Month In Vivo Results from a Juvenile Sheep Model
Source: Eur J Cardiothorac Surg. 2025 Aug 1;67(8):ezaf266. doi: 10.1093/ejcts/ezaf266 (PMC12365636; doi:10.1093/ejcts/ezaf266)
Supplement: ezaf266_Supplementary_Data [file ezaf266_supplementary_data.zip › CORRECT Supplemental file 30 June changes accepted.docx]

**Supplemental file**

**Extended Material and Methods**

**Figure S1**.


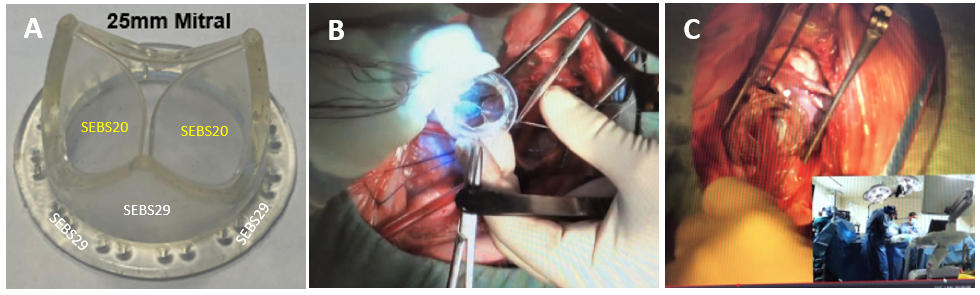


***Open-heart surgical procedure to implant the SEBS29/SEBS20 prototype in mitral position. A****: Polymeric valve made from SEBS29 (more rigid material for sewing skirt and posts) and SEBS20 (more flexible material for leaflets).* ***B****: passing of sutures through premade holes in the skirt.* ***C****: valve sitting on the annulus before sutures being tied off.*

**Table S1. Molecular characterisation**

| Material | Composition | Molecular weight  (g/mol) | PI index | Styrene fraction  (% wt.) |
| --- | --- | --- | --- | --- |
| **SEBS29** | poly(styrene-*block*-ethylene/butylene-*block*-styrene) | 74,800 | 1.06 | 28.4 |
| **SEBS20** | poly(styrene-*block*-ethylene/butylene-*block*-styrene) | 111,300 | 1.07 | 19.6 |

###

### *Premedication, Anaesthesia, Surgery, Postoperative Care and Definitions*

All Study Animals were anesthetized and intubated according to a Standard Operating Procedure (SOP) and operated under general anaesthesia (GA) in line with premedication, induction, maintenance and mechanical ventilation shown in Table S2. Ventilation was fine-tuned during surgery to keep arterial blood gases and PH levels within physiological ranges. A large bore orogastric tube was placed in the rumen to drain fluids by gravity and prevent ruminal distension.

**Table S2** - Synoptic table on animal preparation, anesthesia induction and maintenance, during interim procedures.

| **Premedication** | Morphine 0.20 to 0.22 mg/kg and Midazolam 0.5 mg/kg IM |
| --- | --- |
| **Induction** | Midazolam 0.25 to 0.55 mg/kg IV + Propofol 1.20 to 2.90 mg/ kg IV |
| **Maintenance** | Isoflurane 1 to 5 % or Propofol 8.9 to 19.5 mg/kg/h IV ± Midazolam 0.3 to 0.6 mg/kg/h IV  Respiratory rate 10-20 cycles /min (and according to ETCO_2_)  Volume 10 mL/kg (and according to ETCO_2_) |

During the implantation procedure ECG, blood pressure, end-tidal CO_2_ and core body temperature were monitored and recorded on CRFs as per specific SOP. Cefazolin (20 mg/kg) was administered intravenously (IV) to prevent infections in each study animal just before starting of surgery, every 2 hours for the duration of the intervention, and then IM twice/day for 8 days after surgery.

For surgery, the animals were placed in a right lateral recumbent position, surgically scrubbed, and draped to expose the proximal left cervical region and the left chest separately. Left carotid artery (CA) and jugular vein (JV) were exposed, and left thoracotomy was performed at third intercostal space to expose the heart. Following heparinization to achieve ACT >400 sec, cardiopulmonary bypass (CPB) was established through cannulation of both CA and JV. Once on CPB, adequate flow rate was maintained without cooling. The mitral valve replacement procedure was carried out through an incision made at the left atrial auricle (LAA) to expose the mitral valve, on the vented beating heart with CPB support. The devices were randomly selected at 1:1 ratio from the batch of 6 HC1-6 and 6 N1-6 available and implanted in routine supra-annular fashion using routine 2-0 sutures with 3x3mm Teflon. A valve holder was not available at this early developmental stage hence the prototypes were manually held by the surgical assistant during surgery while the main operator passed the sutures through the set of pre-made holes (Figure S1B). On completion, the left atrium was closed after deairing. With stable hemodynamics, the CPB was weaned off and the CA and JV were decannulated and oversewn. Wounds were closed in layers following positioning of a chest drain.

After surgery, animals were continuously observed in the recovery area until able to breathe comfortably and demonstrating stable vital signs as determined by the Study Director or a deputy or an Animal Health Technician. Once stable, each Study Animal was observed hourly for the next 24. All observations were recorded on the CRF. For postoperative analgesia, Morphine 0.2 mg/kg IM was administered twice/day on Day 1 and Day 2 in all Study Animals and Meloxicam 0.5 mg/kg IM was administered once/day on Day 1 and Day 2. No vitamin K antagonists were used. Instead, animals received Enoxaparin 1 mg/kg SC twice/day from Day 1 until Day 120 and Clopidogrel 300 mg with food once/day from Day 1 until sacrifice. Serial anticoagulation screening was undertaken to document the real levels of anticoagulation obtained with the used dosage of enoxaparin over the study period. Levels of platelet inhibition were not tested. Diuretics (Furosemide) were given IM twice per day from Day 1 until sacrifice if > mild mitral regurgitation was present after surgery*.* All medications given postoperatively were documented in the CRF. For animal welfare reasons, the Study Director or a Deputy could change the type of drugs, dosage or duration in line with routine veterinary practice. Any changes would not be regarded as Protocol Deviation and would be documented in the CRF.

Any postoperative complications (e.g., bleeding, thromboembolism, stroke, infections, endocarditis, or others) were reported to the Study Director for assessment/confirmation and recorded in the CRF. In case of signs of severe debility, particularly if death appeared imminent, animals were electively sacrificed according to a predefined SOP if unable to feed and/or ambulate or if in class IV NYHA heart failure despite adequate treatment. This was done for humane reasons and to prevent the loss of tissue through autolysis. Early death was defined as any study animal dying before the scheduled 180±5 days termination date. Non-material related sacrifice or death were defined as events not related to the material used but as consequences of the surgical procedure, if clinically indicated.

**Assessment of Material Safety**

*Necroscopy*

After culling a gross examination was performed by the Study Director, or Deputy or appointed Staff Veterinarian or the Study Pathologist as per specific SOP*.* The study animals were observed carefully for external abnormalities including palpable masses. The abdominal and thoracic cavities were examined for abnormalities and the target tissues removed, examined, and where required, whole organs or tissue samples were be placed in 10 % neutral buffered formalin (approximately 4% formaldehyde solution) jars labelled with Study number, Study Animal number, explant day, explant date and sample identification as per specific SOP*.* The heart was harvested with the Test Article *in situ*, weighed, assessed macroscopically, and photographed as per specific SOPs*.* High-resolution X-ray (Faxitron®) of the Test Article *in situ* was performed for assessment of calcification. Sections of the Test Article were then obtained and submitted for histopathology evaluation*.* Standard samples of the heart were collected and systematically placed in 10 % neutral buffered formalin (approximately 4% formaldehyde solution) jars labelled with Study number, Study Animal number, explant day, explant date and sample identification as per specific SOP*.* Standard samples of the lungs, liver, spleen, kidneys, adrenal glands, regional lymph nodes and brain were collected and systematically placed in 10 % neutral buffered formalin (approximately 4% formaldehyde solution) jars labelled with Study number, Study Animal number, explant day, explant date and sample identification as per specific SOP***.*** Any additional abnormal macroscopic finding was also photographed and sampled at the discretion of the Study Director or Study Pathologist for histopathological assessment.

*Material degradation*

*Gel Permeation Chromatography (GPC)/Size Exclusion Chromatography (SEC).*

GPC/SEC was carried out on the Viscotek GPCmax (Malvern Panalytical), equipped with 270 dual detector system, Viscotek UV detector 2500 and RI detector VE 3580. About 10 mg of SEBS29 polymer has been cut from each explanted valve and dissolved in tetrahydrofuran (THF) GPC grade (Fisher Scientific), to form an eluent of 1 mg/ml concentration. A defined volume of the sample solution has been filtered and loaded onto the column using an autosampler. The flow rate was kept at 1ml min^-1^. The separation column was LT5000L, Mixed, Medium Org, packed with microparticulate material (styrene-divinylbenzene gel) of 10 µm particle size. The principle of GPC/SEC is the separation of molecules based on their hydrodynamic radius or volume. The larger molecules are excluded from some of the pores in the packing material and therefore elute faster through the column than the smaller molecules. In effect, the molecules are separated by size, with the largest eluting first and the smallest last. When the sample elutes from the column it passes through a series of detectors and the output is analysed by a GPC/SEC software package on the computer. The triple detection available in the Viscotek instrument employs concentration detectors (UV and RI) which are necessary for the determination of both molecular weight and intrinsic viscosity whereas the viscometer detector provides a direct measurement of intrinsic viscosity or molecular density to provide information on the structure. In addition, the light scattering detector provides a direct measurement of absolute molecular weight. Such a combination provides complementary but different characteristics. Three samples from each valve were taken for the analyses. Furthermore, the sample taken from the valve being implanted into sheep for only 24 hours (11684) as well as the raw SEBS29 material (G1650) were measured in the same system for reference.

*Calcification of cardiac tissue directly interfacing with SEBS29 material*

This was carried out in the Ascione’s lab on fixed heart specimens with embedded SEBS29 material received from the testing facility. Myocardial mitral annular tissue from all 6 study animals surviving surgery was sampled to include tissue directly interfacing with SEBS29 or adjacent but not interfacing tissue (2mm away) for control. Tissue samples were embedded in paraffin using a standard technique. Microscope slides were prepared with 5-µm tissue sections. Slides were deparaffinized with a xylene substitute, rehydrate by decreasing ethanol concentrations and stained with haematoxylin and eosin (H&E) for cell number quantification and von Kossa staining for calcification. For cell number quantification the number of nuclei was manually counted do derive the number of cells present in each study animal across 3 regions of interest (ROIs): the zone directly interfacing with SEBS29 and two adjacent control regions 2mm away. A variable number of ROIs was determined for a total coverage of the zone directly interfacing with SEBS29 and the control areas for each sample (nPics). The nuclei number was determined at magnification X20 on pictures obtained by M8 Microscope and Scanner. ImageJ software was used for manual nuclei quantification. For assessment of calcification von Kossa staining was used. The same ROIs tissue sections were incubated in 1% silver nitrate. Ultra-violet light was applied for 20 minutes, with washing with a solution of 5% sodium thiosulfate for 5 minutes and counterstaining with nuclear fast red for 5 minutes. Visual inspection of the images was performed. Magnification X40 scan files were obtained of the entire tissue sections by M8 Microscope and Scanner and visual inspections were performed. Positive controls were obtained by culturing millimetric sections of human saphenous vein in High Glucose DMEM supplemented with 10% foetal bovine serum, 100 U/ml penicillin/streptomycin, and 2.5 mM L-glutamine in the presence of 10 mM β-glycerophosphate, 8 mM CaCl2 and 50 µM ascorbic acid for 7 day^1^. With this approach the presence of calcium was highlighted by black stained precipitate following von Kossa staining.

**Results**

The case of non-material related early death occurred on day 81 and was due to a late valve malfunction associated with one of the surgical sutures looping around one of the 3 valve posts blocking it and leading to late associated tears (Figure S2).

**Figure S2**


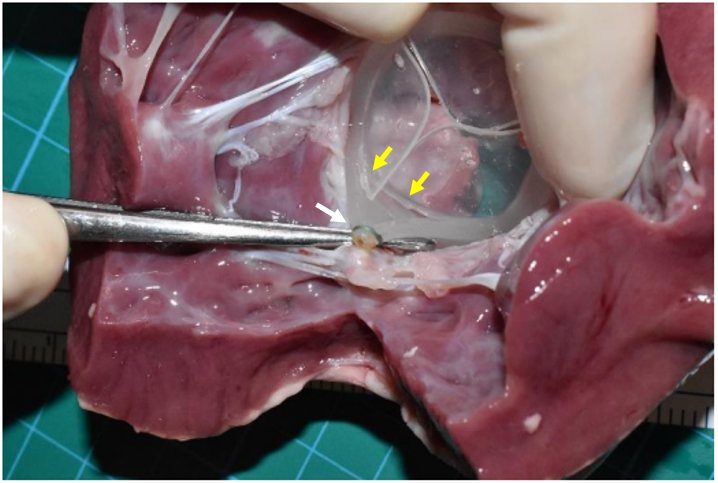


*Outflow view of the Test Article for Study Animal 02448 suffering a non-material related death on day 81. One of the surgical sutures is seen looping around one of the 3 valve posts blocking it (white arrow) with associated tears at the SEBS29/SEBS20 interface (yellow arrows).*

**Figure S3**

.


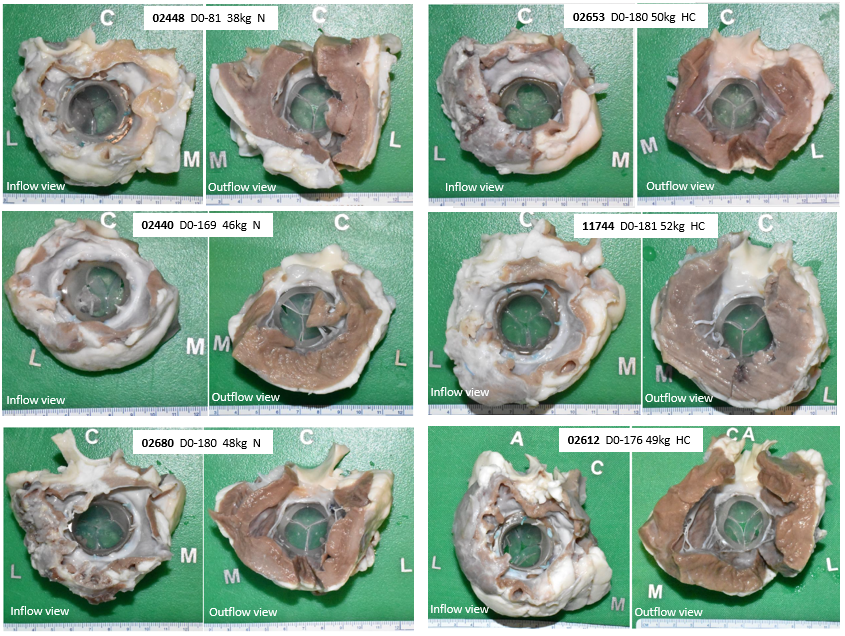


***Macroscopy examination.*** *Photographs of the in-situ prototypes showing inflow and outflow views of all 6 prototypes with skirt/ring (SEBS29) and the leaflets (SEBS20) clearly seen. The legend for each prototype shows study animal, termination day, animal weight at surgery and N (normal) or HC (heparin-coated) sub-groups. L: Lateral cusp; C: Cranial cusp; M: Medial cusp.*

*Material degradation*

We found no evidence of degradation in the SEBS29 material samples explanted at 6-month compared to the SEBS29 sample explanted after 24 hours (11684) and to the raw SEBS29 sample (G1650). All tested SEBS29 samples generated nearly identical GPC elution curves as shown by the three detectors in the chromatography system (Table S3). The slight variation in the peak height of the curves is associated with some small changes in the concentration of the analysed samples. They all however showed the maximum of the elution profile at the same retention volume which is the amount of liquid that passed from the point of injection until the response of a detector. As the samples were run at a constant flow rate of 1 ml min^-1^, the retention volume was equal to the retention time in minutes. The GPC by size exclusion separates polymer chains by their molecular size. The largest size molecules exit the column first with smaller molecules exiting incrementally later as size decreases. The average retention time for the maximum signal intensity was 16.7 minutes (Table S4). This suggests that the average molecular weight of the explanted samples and the reference samples were the same. This in turn indicates that the explanted samples have not degraded and did not break into shorter chains during the 6-month sheep trial. Table S3 contains the detailed molecular characteristics like the average molecular weight (Mw), molecular number (Mn), polydispersity (PI) and the retention time for the explanted samples and the control samples. Multiple parameters intervening in these complex data processing can influence the reproducibility of results between replicates in many ways.

**Table S4** Molecular characterisation of the explanted and raw SEBS29 samples.

|  | 02440-A | 02448_a | 02612-A | 02653-A | 02680-A | 11744-A | 11684-C | G1650 |
| --- | --- | --- | --- | --- | --- | --- | --- | --- |
| M_W_  (kDa) | 73.4 *±3.6* | 76.3 *±4.4* | 76.2 *±0.7* | 74.4 *±0.3* | 75.1 *±2.4* | 72.8 *±0.4* | 75.4*±5.9* | 75.4*±0.5* |
| M_n_ (kDa) | 65.2 *±4.1* | 66.6 *±4.8* | 67.3 *±1.9* | 63.6 *±0.4* | 66.3 *±1.9* | 63.6 *±2.5* | 66.9 *±8.8* | 66.7*±2.6* |
| PI | 1.13 *±0.02* | 1.15 *±0.02* | 1.13 *±0.03* | 1.13 *±0.02* | 1.17 *±0.01* | 1.14 *±0.04* | 1.13 *±0.06* | 1.13 *±0.04* |
| Ret.time(min) | 16.7 *±0.02* | 16.7 *±0.02* | 16.7 *±0.02* | 16.7 *±0.01* | 16.7 *±0.01* | 16.7 *±0.01* | 16.7 *±0.01* | 16.7 *±0.02* |

*Calcification of cardiac tissue directly interfacing with SEBS29 material*

**Figure S4**

**
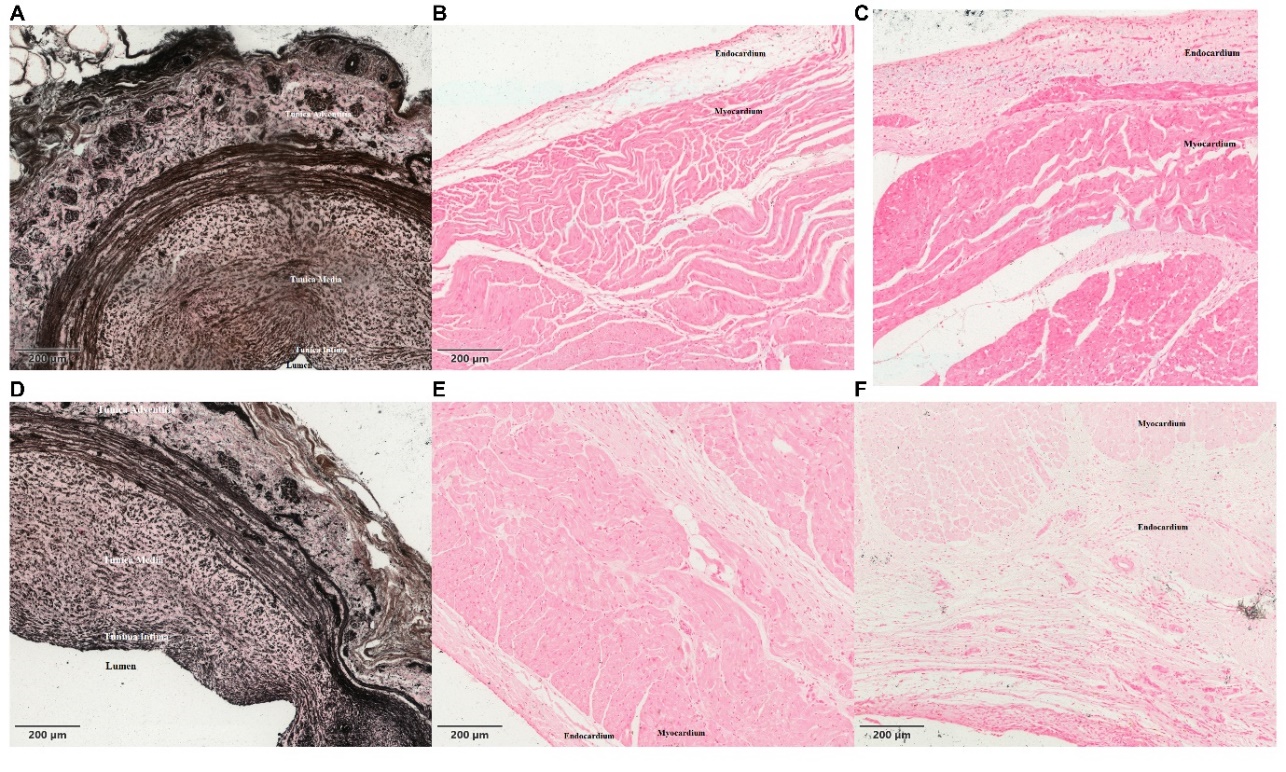
**

**Calcification of cardiac tissue interfacing SEBS29** (*von Kossa staining showing calcium deposits in black)*: ***A*** *and* ***D****: positive control calcified human saphenous vein.* ***B*** *and* ***C****: tissue samples from the animal culled on day 2;* ***B****=myocardial tissue away from SEBS29;* ***C****=myocardial tissue in contact with SEBS29; at 1.25X and 2X magnification levels, respectively.* ***E*** *and* ***F****: tissue samples from a representative animal culled on day 180;* ***E****=myocardial tissue away SEBS29;* ***F****= myocardial tissue in contact with SEBS29; at 1.5X and 0.6X magnification levels, respectively.*

**Figure S5**


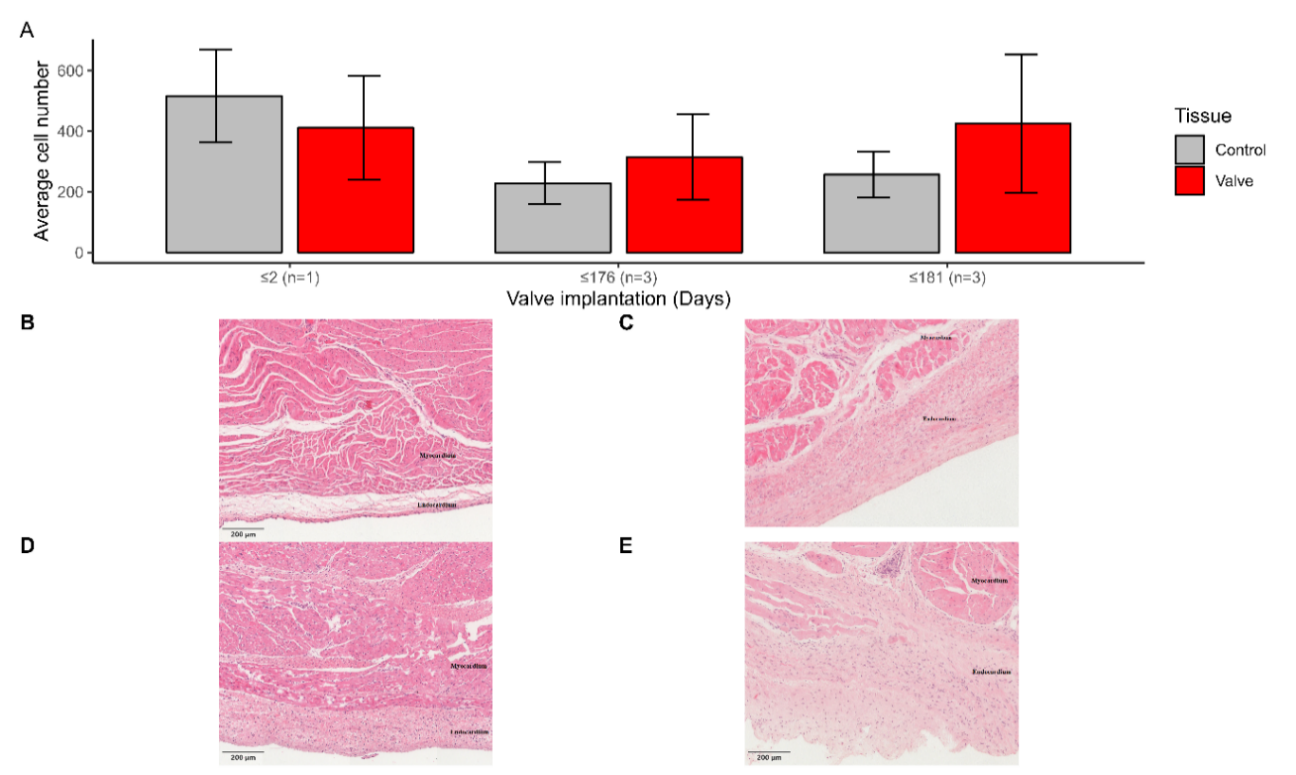


**Histology of cardiac tissue interfacing SEBS29.** ***A****: bar graphic showing the average number of cell nuclei at the myocardial area directly interfacing with SEBS29 (red box) and control region (grey box).* ***B-C****: 10X amplification of control myocardial tissue at day 2 and 169;* ***D-E****: 10X amplifiaction of myocardial tissue in direct contact with SEBS29 at day 2 and 169.*

**Table S5 -** Cell numbers across study animals and overtime

| ID | Time | Tissue | nPics | Cells | SD | Time | n | Tissue | Mean n.  Cells | SD |
| --- | --- | --- | --- | --- | --- | --- | --- | --- | --- | --- |
| 1_11684 | 2 | Control | 6 | 516.33 | 152.78 | ≤2 (n=1) | 1 | Control | 516.33 | 152.78 |
| 1_11684 | 2 | SEBS29 | 16 | 411.56 | 170.37 | ≤2 (n=1) |  | Valve | 411.56 | 170.37 |
| 5_02448 | 81 | Control | 16 | 256.56 | 87.73 |  |  |  |  |  |
| 5_02448 | 81 | Sebs29 | 24 | 346.38 | 149.46 |  |  |  |  |  |
| 3_02440 | 169 | Control | 7 | 151.71 | 46.64 |  |  |  |  |  |
| 3_02440 | 169 | Valve | 16 | 317.44 | 157.22 |  |  |  |  |  |
| 4_02612 | 176 | Control | 8 | 277.00 | 52.63 | ≤176 (n=3) | 3 | Control | 228.43 | 69.07 |
| 4_02612 | 176 | Valve | 18 | 280.17 | 125.28 | ≤176 (n=3) |  | Valve | 314.66 | 140.89 |
| 6_02680 | 180 | Control | 16 | 249.00 | 68.13 |  |  |  |  |  |
| 6_02680 | 180 | Valve | 30 | 402.67 | 274.20 |  |  |  |  |  |
| 7_02653 | 180 | Control | 16 | 245.19 | 80.38 |  |  |  |  |  |
| 7_02653 | 180 | Valve | 23 | 333.43 | 184.62 |  |  |  |  |  |
| 2_11744 | 181 | Control | 16 | 277.31 | 82.81 | ≤181 (n=3) | 3 | Control | 257.17 | 74.92 |
| 2_11744 | 181 | Valve | 16 | 537.88 | 208.85 | ≤181 (n=3) |  | Valve | 424.66 | 227.92 |
| *ID: Identification code; Time: Duration of in-vivo implantation (days); Tissue = Control: tissue samples not interfacing with material and SEBS29: samples directly in contact with material; Cells: number of nuclei, Average Cells: average number of nuclei of samples per time group, Average SD: Average of SD of nuclei of samples per time group; Average S.D.* | | | | | | | | | | |

**Figure S6**


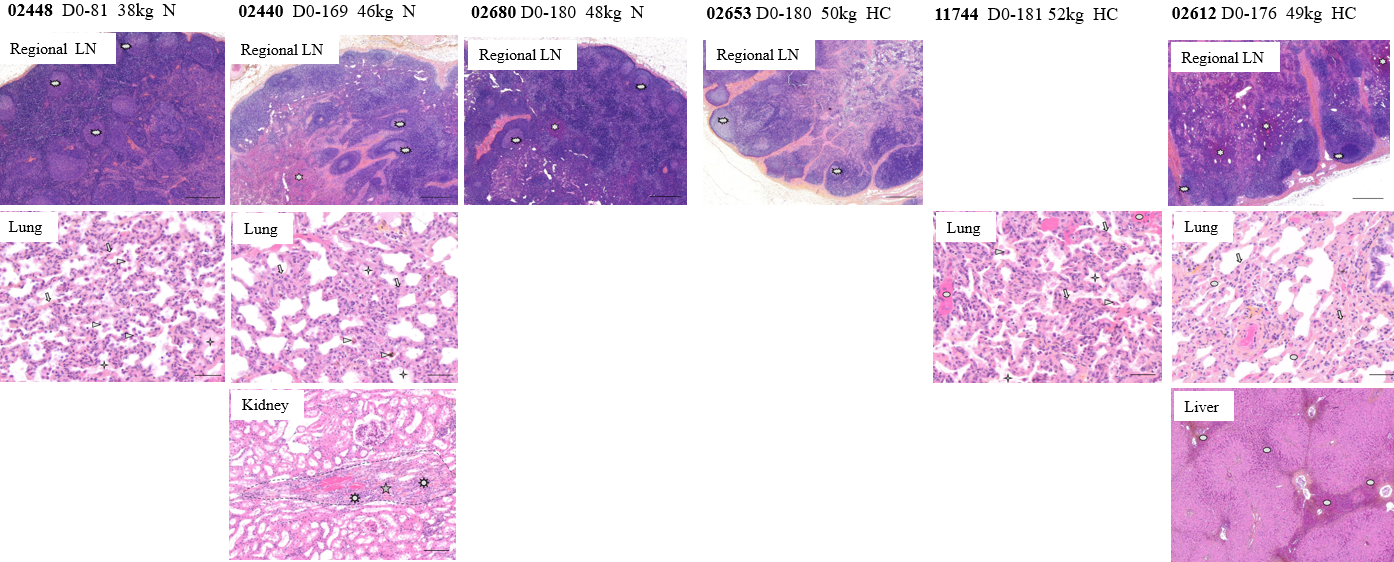


*
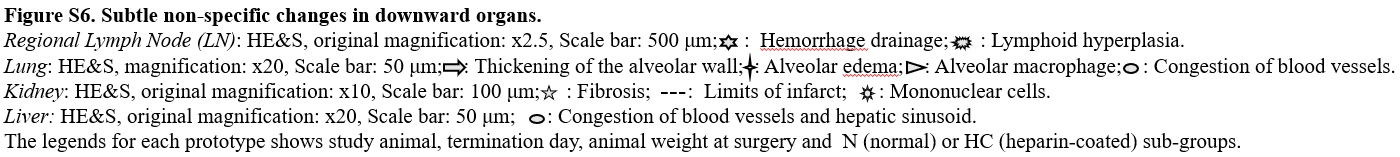
*

*Clinical Pathology*

**Table S6 - Clinical Pathology of all cases**

|  | Study Animal #02440 | | | | | | Reference Interval | | Units |  |
| --- | --- | --- | --- | --- | --- | --- | --- | --- | --- | --- |
|  | Day 0 | Day 14 | Day 30 | Day 81 | Day 120 | Day 169 | Low | High |  |  |
| **Urea** | 5.30 | 7.10 | 5.20 | 8.30 | 7.50 | NAv | *2.30* | *10.20* | *mmol/L* | |
| **Creatinine** | 117.00 | 85.00 | 110.00 | 93.00 | 105.00 | NAv | *68.00* | *150.00* | *µmol/L* | |
| **Glucose** | 3.80 | 4.10 | 4.20 | 4.20 | 3.90 | NAv | *2.40* | *9.50* | *mmol/L* | |
| **Sodium** | 146.00 | 145.00 | 142.00 | 144.00 | 143.00 | NAv | *142.00* | *155.50* | *mmol/L* | |
| **Potassium** | 5.40 | 4.70 | 4.80 | 4.40 | 4.40 | NAv | *3.90* | *6.30* | *mmol/L* | |
| **Chloride** | 108.00 | 102.00 | 101.00 | 100.00 | 100.00 | NAv | *98.00* | *112.00* | *mmol/L* | |
| **Bicarbonate** | 28.20 | 31.50 | 29.50 | **32.30** | 31.90 | NAv | *19.00* | *32.00* | *mmol/L* | |
| **Plasma Proteins** | 78.00 | 71.60 | 73.60 | 66.00 | 68.80 | NAv | *60.00* | *86.00* | *g/L* | |
| **Calcium** | 2.65 | 2.65 | 2.62 | 2.59 | 2.61 | NAv | *2.02* | *2.81* | *mmol/L* | |
| **Phosphorus** | 2.00 | 2.71 | 2.78 | **3.93** | 2.36 | NAv | *1.09* | *3.27* | *mmol/L* | |
| **Magnesium** | 0.85 | 0.86 | 0.89 | 0.89 | 0.87 | NAv | *0.64* | *1.11* | *mmol/L* | |
| **Total Bilirubin** | <3 | <3 | <3 | <3 | <3 | NAv | *0.00* | *6.00* | *µmol/L* | |
| **Alkaline Phosphatase** | 97.00 | 81.00 | 89.00 | 193.00 | 152.00 | NAv | *37.00* | *299.00* | *U/L* | |
| **Gamma Glutamyl Transferases** | 58.00 | 67.00 | 53.00 | 63.00 | 64.00 | NAv | *33.00* | *84.00* | *U/L* | |
| **AST** | 104.00 | 92.00 | 89.00 | 123.00 | NAv | NAv | *67.50* | *253.50* | *U/L* | |
| **ALT** | 19.00 | 17.00 | 23.00 | 23.00 | 33.00 | NAv | *9.00* | *46.50* | *U/L* | |

### Study Animal 02440 ***-*** Biochemistry results - *NAv = not available

### Study Animal 02440 - Plasma Free Haemoglobin results

|  | **Study Animal #02440** | | | | | | Reference Interval | | Units |
| --- | --- | --- | --- | --- | --- | --- | --- | --- | --- |
|  | Day 0 | Day 14 | Day 30 | Day 81 | Day 120 | Day 169 | Low | High |  |
| **Plasma Free Hemoglobin** | 0.30 | 0.30 | 0.50 | 0.30 | 0.50 | 0.40 | *0.00* | *1.04* | *g/L* |

|  | **Study Animal #02440** | | | | | | Reference Interval | | Units |
| --- | --- | --- | --- | --- | --- | --- | --- | --- | --- |
|  | Day 0 | Day 14 | Day 30 | Day 81 | Day 120 | Day 169 | Low | High |  |
| **RBC** | 13.90 | 10.60 | 12.00 | 11.20 | 10.60 | 9.00 | *9.10* | *14.70* | *10^12^/L* |
| **Hemoglobin** | 12.70 | 10.30 | 11.90 | 11.40 | 10.80 | **8.90** | *9.60* | *14.80* | *g/dL* |
| **Hematocrit** | 0.37 | 0.30 | 0.34 | 0.31 | 0.29 | **0.26** | *0.27* | *0.43* | *L/L* |
| **MCV** | 27.00 | 28.00 | 28.00 | 28.00 | 27.00 | 29.00 | *25.00* | *35.00* | *fL* |
| **MCHC** | 34.00 | 34.00 | 35.00 | 37.00 | 37.00 | 34.00 | *31.00* | *39.00* | *g/dL* |
| **MCH** | 9.00 | 10.00 | 10.00 | 10.00 | 10.00 | 10.00 | *9.00* | *12.00* | *pg* |
| **WBC** | 7.00 | 6.80 | 8.30 | 7.70 | 6.10 | 4.10 | *3.40* | *10.80* | *10^9^/L* |
| ***Neutrophil ratio*** | *38.00* | *34.00* | *31.00* | *26.00* | *32.00* | *24.00* | *21.00* | *64.00* | *%* |
| ***Eosinophil ratio*** | *3.00* | *12.00* | *20.00* | *10.00* | *5.00* | *4.00* | *1.00* | *19.00* | *%* |
| ***Basophil ratio*** | *1.00* | *0.00* | *1.00* | *1.00* | *1.00* | *1.00* | *0.00* | *2.00* | *%* |
| ***Lymphocyte ratio*** | *56.00* | *53.00* | *47.00* | *60.00* | *61.00* | *69.00* | *30.00* | *71.00* | *%* |
| ***Monocyte ratio*** | *2.00* | *1.00* | *1.00* | *3.00* | *1.00* | *2.00* | *1.00* | *9.00* | *%* |
| **Platelets** | 704.00 | **858.00** | 751.00 | 660.00 | 470.00 | 468.00 | *119.00* | *765.00* | *10^9^/L* |
| **Reticulocytes** | 0.20 | 0.20 | 0.35 | 0.10 | 0.20 | 0.17 | *0.03* | *0.36* | *%* |

Study Animal 02440 - CBC results

Study Animal #02440 Coagulation results. *NAv = not available

|  | **Study Animal #02440** | | | | | | Reference Interval | | Units |
| --- | --- | --- | --- | --- | --- | --- | --- | --- | --- |
|  | Day 0 | Day 14 | Day 30 | Day 81 | Day 120 | Day 169 | Low | High |  |
| **PR** | **24.00** | **29.00** | **25.00** | **27.00** | **24.00** | NAv | *33.00* | *82.00* | *%* |
| **aPTT** | 30.30 | 29.90 | 31.40 | 32.10 | 29.40 | NAv | *22.00* | *44.00* | *sec* |
| **Fibrinogen** | 2.70 | 1.60 | 1.80 | 1.80 | 2.00 | NAv | *1.50* | *4.90* | *g/L* |

Study Animal 02448 - Biochemistry results.

|  | **Study Animal #02448** | | | | Reference Interval | | Units |
| --- | --- | --- | --- | --- | --- | --- | --- |
|  | Day 0 | Day 14 | Day 30 | Day 81 | Low | High |  |
| **Urea** | 6.00 | 7.80 | 7.30 | 8.30 | *2.30* | *10.20* | *mmol/L* |
| **Creatinine** | 98.00 | 89.00 | 104.00 | 94.00 | *68.00* | *150.00* | *µmol/L* |
| **Glucose** | 3.50 | 4.30 | 3.70 | 4.00 | *2.40* | *9.50* | *mmol/L* |
| **Sodium** | 146.00 | 146.00 | 147.00 | 149.00 | *142.00* | *155.50* | *mmol/L* |
| **Potassium** | 4.40 | 4.30 | 5.80 | **3.80** | *3.90* | *6.30* | *mmol/L* |
| **Chloride** | 109.00 | 104.00 | 107.00 | 102.00 | *98.00* | *112.00* | *mmol/L* |
| **Bicarbonate** | 27.70 | 31.20 | 30.20 | **36.70** | *19.00* | *32.00* | *mmol/L* |
| **Plasma Proteins** | 76.10 | 66.40 | 71.90 | 66.10 | *60.00* | *86.00* | *g/L* |
| **Calcium** | 2.50 | 2.51 | 2.44 | 2.42 | *2.02* | *2.81* | *mmol/L* |
| **Phosphorus** | 1.95 | 2.85 | 3.25 | **3.29** | *1.09* | *3.27* | *mmol/L* |
| **Magnesium** | 0.85 | 0.76 | 0.88 | 0.86 | *0.64* | *1.11* | *mmol/L* |
| **Total Bilirubin** | <3 | <3 | <3 | <3 | *0.00* | *6.00* | *µmol/L* |
| **Alkaline Phosphatase** | 100.00 | 110.00 | 125.00 | 96.00 | *37.00* | *299.00* | *U/L* |
| **Gamma Glutamyl Transferases** | 52.00 | 58.00 | 52.00 | 59.00 | *33.00* | *84.00* | *U/L* |
| **AST** | 75.00 | 77.00 | 82.00 | 89.00 | *67.50* | *253.50* | *U/L* |
| **ALT** | 11.00 | 16.00 | 16.00 | 13.00 | *9.00* | *46.50* | *U/L* |

Study Animal 02448 *-* Plasma Free Haemoglobin results.

|  | **Study Animal #02448** | | | | Reference Interval | | Units |
| --- | --- | --- | --- | --- | --- | --- | --- |
|  | Day 0 | Day 14 | Day 30 | Day 81 | Low | High |  |
| **Plasma Free Hemoglobin** | 0.30 | 0.30 | 0.50 | 0.30 | *0.00* | *1.04* | *g/L* |

Study Animal 02448 *-* CBC results.

|  | **Study Animal #02448** | | | | Reference Interval | | Units |
| --- | --- | --- | --- | --- | --- | --- | --- |
|  | Day 0 | Day 14 | Day 30 | Day 81 | Low | High |  |
| **RBC** | 13.00 | 9.80 | 12.20 | 12.20 | *9.10* | *14.70* | *10^12^/L* |
| **Hemoglobin** | 11.70 | 9.90 | 12.00 | 12.20 | *9.60* | *14.80* | *g/dL* |
| **Hematocrit** | 0.34 | 0.30 | 0.36 | 0.34 | *0.27* | *0.43* | *L/L* |
| **MCV** | 26.00 | 31.00 | 30.00 | 28.00 | *25.00* | *35.00* | *fL* |
| **MCHC** | 34.00 | 33.00 | 33.00 | 36.00 | *31.00* | *39.00* | *g/dL* |
| **MCH** | 9.00 | 10.00 | 10.00 | 10.00 | *9.00* | *12.00* | *pg* |
| **WBC** | 8.60 | 8.90 | 8.80 | 8.60 | *3.40* | *10.80* | *10^9^/L* |
| ***Neutrophil ratio*** | *41.00* | *46.00* | *36.00* | *43.00* | *21.00* | *64.00* | *%* |
| ***Eosinophil ratio*** | *1.00* | *4.00* | *6.00* | *5.00* | *1.00* | *19.00* | *%* |
| ***Basophil ratio*** | *0.00* | *0.00* | *0.00* | *0.00* | *0.00* | *2.00* | *%* |
| ***Lymphocyte ratio*** | *56.00* | *49.00* | *52.00* | *51.00* | *30.00* | *71.00* | *%* |
| ***Monocyte ratio*** | *2.00* | *1.00* | *6.00* | *1.00* | *1.00* | *9.00* | *%* |
| **Platelets** | 697.00 | 566.00 | 681.00 | 422.00 | *119.00* | *765.00* | *10^9^/L* |
| **Reticulocytes** | 0.22 | **0.59** | 0.24 | 0.15 | *0.03* | *0.36* | *%* |

Study Animal 02448 - Coagulation results.

|  | **Study Animal #02448** | | | | Reference Interval | | Units |
| --- | --- | --- | --- | --- | --- | --- | --- |
|  | Day 0 | Day 14 | Day 30 | Day 81 | Low | High |  |
| **PR** | **24.00** | **26.00** | **24.00** | **24.00** | *33.00* | *82.00* | *%* |
| **aPTT** | 28.90 | 35.60 | 34.00 | 37.70 | *22.00* | *44.00* | *sec* |
| **Fibrinogen** | 3.90 | 1.70 | **1.40** | **1.40** | *1.50* | *4.90* | *g/L* |

Study Animal 11744 - Biochemistry results.

|  | **Study Animal #11744** | | | | | Reference Interval | | Units |
| --- | --- | --- | --- | --- | --- | --- | --- | --- |
|  | Day 0 | Day 14 | Day 30 | Day 120 | Day 181 | Low | High |  |
| **Urea** | 6.80 | 7.20 | 6.90 | 7.00 | 7.80 | *2.30* | *10.20* | *mmol/L* |
| **Creatinine** | 112.00 | 89.00 | 101.00 | 110.00 | 99.00 | *68.00* | *150.00* | *µmol/L* |
| **Glucose** | 5.70 | 4.30 | 4.00 | 4.10 | 7.40 | *2.40* | *9.50* | *mmol/L* |
| **Sodium** | 146.00 | 146.00 | 146.00 | 146.00 | 147.00 | *142.00* | *155.50* | *mmol/L* |
| **Potassium** | 4.30 | 5.30 | 5.00 | 5.60 | **3.50** | *3.90* | *6.30* | *mmol/L* |
| **Chloride** | 107.00 | 104.00 | 105.00 | 105.00 | **96.00** | *98.00* | *112.00* | *mmol/L* |
| **Bicarbonate** | 29.10 | **32.60** | 29.50 | 26.40 | **36.60** | *19.00* | *32.00* | *mmol/L* |
| **Plasma Proteins** | 69.80 | 64.80 | 65.00 | 67.50 | 73.40 | *60.00* | *86.00* | *g/L* |
| **Calcium** | 2.21 | 2.48 | 2.43 | 2.35 | 2.39 | *2.02* | *2.81* | *mmol/L* |
| **Phosphorus** | 2.54 | 2.94 | 2.99 | 3.09 | 3.07 | *1.09* | *3.27* | *mmol/L* |
| **Magnesium** | 0.80 | 0.88 | 0.88 | 0.95 | 0.78 | *0.64* | *1.11* | *mmol/L* |
| **Total Bilirubin** | <3 | <3 | <3 | <3 | 4.00 | *0.00* | *6.00* | *µmol/L* |
| **Alkaline Phosphatase** | 190.00 | 177.00 | 196.00 | **311.00** | 247.00 | *37.00* | *299.00* | *U/L* |
| **Gamma Glutamyl Transferases** | 49.00 | 62.00 | 55.00 | 56.00 | 72.00 | *33.00* | *84.00* | *U/L* |
| **AST** | 117.00 | 104.00 | 109.00 | 153.00 | 156.00 | *67.50* | *253.50* | *U/L* |
| **ALT** | 28.00 | 24.00 | 29.00 | 39.00 | 37.00 | *9.00* | *46.50* | *U/L* |

Study Animal 11744 - Plasma Free Haemoglobin results.

|  | **Study Animal #11744** | | | | | Reference Interval | | Units |
| --- | --- | --- | --- | --- | --- | --- | --- | --- |
|  | Day 0 | Day 14 | Day 30 | Day 120 | Day 181 | Low | High |  |
| **Plasma Free Hemoglobin** | 0.30 | 0.60 | 0.30 | 0.60 | 0.20 | *0.00* | *1.04* | *g/L* |

Study Animal 11744 - CBC results.

|  | **Study Animal #11744** | | | | | Reference Interval | | Units |
| --- | --- | --- | --- | --- | --- | --- | --- | --- |
|  | Day 0 | Day 14 | Day 30 | Day 120 | Day 181 | Low | High |  |
| **RBC** | 11.00 | 9.50 | 10.40 | 11.20 | 11.10 | *9.10* | *14.70* | *10^12^/L* |
| **Hemoglobin** | 11.80 | 10.40 | 11.80 | 12.70 | 12.40 | *9.60* | *14.80* | *g/dL* |
| **Hematocrit** | 0.36 | 0.33 | 0.37 | 0.38 | 0.37 | *0.27* | *0.43* | *L/L* |
| **MCV** | 33.00 | 35.00 | **36.00** | 34.00 | 33.00 | *25.00* | *35.00* | *fL* |
| **MCHC** | 33.00 | 32.00 | 32.00 | 33.00 | 34.00 | *31.00* | *39.00* | *g/dL* |
| **MCH** | 11.00 | 11.00 | 11.00 | 11.00 | 11.00 | *9.00* | *12.00* | *pg* |
| **WBC** | 4.20 | 4.10 | 4.60 | 5.00 | 3.90 | *3.40* | *10.80* | *10^9^/L* |
| ***Neutrophil ratio*** | ***17.00*** | *33.00* | *24.00* | *25.00* | *42.00* | *21.00* | *64.00* | *%* |
| ***Eosinophil ratio*** | *3.00* | *2.00* | *2.00* | *2.00* | *3.00* | *1.00* | *19.00* | *%* |
| ***Basophil ratio*** | *1.00* | *0.00* | *1.00* | *0.00* | *0.00* | *0.00* | *2.00* | *%* |
| ***Lymphocyte ratio*** | ***75.00*** | *61.00* | *69.00* | *71.00* | *54.00* | *30.00* | *71.00* | *%* |
| ***Monocyte ratio*** | *4.00* | *4.00* | *4.00* | *2.00* | *1.00* | *1.00* | *9.00* | *%* |
| **Platelets** | 481.00 | 680.00 | 545.00 | 428.00 | 406.00 | *119.00* | *765.00* | *10^9^/L* |
| **Reticulocytes** | 0.16 | 0.33 | 0.21 | 0.12 | 0.13 | *0.03* | *0.36* | *%* |

Study Animal 11744 - Coagulation results.

|  | **Study Animal #11744** | | | | | Reference Interval | | Units |
| --- | --- | --- | --- | --- | --- | --- | --- | --- |
|  | Day 0 | Day 14 | Day 30 | Day 120 | Day 181 | Low | High |  |
| **PR** | **27.00** | 34.00 | **32.00** | **27.00** | **32.00** | *33.00* | *82.00* | *%* |
| **aPTT** | 34.80 | 33.10 | 34.10 | 33.20 | 28.10 | *22.00* | *44.00* | *sec* |
| **Fibrinogen** | 2.40 | 1.80 | **1.40** | 1.50 | 2.90 | *1.50* | *4.90* | *g/L* |

Study Animal 02612 - Biochemistry results.

|  | **Study Animal #02612** | | | | | Reference Interval | | Units |
| --- | --- | --- | --- | --- | --- | --- | --- | --- |
|  | Day 0 | Day 15 | Day 29 | Day 119 | Day 176 | Low | High |  |
| **Urea** | 5.90 | 5.60 | 5.90 | 6.20 | 5.80 | *2.30* | *10.20* | *mmol/L* |
| **Creatinine** | 129.00 | 110.00 | 113.00 | 104.00 | 129.00 | *68.00* | *150.00* | *µmol/L* |
| **Glucose** | 4.20 | 4.50 | 3.90 | 5.40 | 7.10 | *2.40* | *9.50* | *mmol/L* |
| **Sodium** | 145.00 | 148.00 | 147.00 | 152.00 | 147.00 | *142.00* | *155.50* | *mmol/L* |
| **Potassium** | 4.50 | 5.00 | 4.40 | 5.00 | **3.10** | *3.90* | *6.30* | *mmol/L* |
| **Chloride** | 112.00 | 108.00 | 106.00 | 109.00 | 102.00 | *98.00* | *112.00* | *mmol/L* |
| **Bicarbonate** | 26.50 | 28.60 | 29.10 | 28.90 | **36.00** | *19.00* | *32.00* | *mmol/L* |
| **Plasma Proteins** | 74.20 | 67.60 | 65.90 | 64.20 | 66.50 | *60.00* | *86.00* | *g/L* |
| **Calcium** | 2.54 | 2.55 | 2.67 | 2.56 | **2.93** | *2.02* | *2.81* | *mmol/L* |
| **Phosphorus** | 1.94 | 2.85 | 2.50 | 2.51 | 1.91 | *1.09* | *3.27* | *mmol/L* |
| **Magnesium** | 1.04 | 1.07 | 0.94 | 0.92 | 0.76 | *0.64* | *1.11* | *mmol/L* |
| **Total Bilirubin** | <3 | <3 | <3 | <3 | <3 | *0.00* | *6.00* | *µmol/L* |
| **Alkaline Phosphatase** | 130.00 | 131.00 | 170.00 | 168.00 | 53.00 | *37.00* | *299.00* | *U/L* |
| **Gamma Glutamyl Transferases** | 50.00 | 52.00 | 47.00 | 51.00 | **88.00** | *33.00* | *84.00* | *U/L* |
| **AST** | 80.00 | 71.00 | **64.00** | 83.00 | 75.00 | *67.50* | *253.50* | *U/L* |
| **ALT** | 19.00 | 10.00 | 14.00 | 18.00 | 9.00 | *9.00* | *46.50* | *U/L* |

Study Animal 02612 - Plasma Free Hemoglobin results.

|  | **Study Animal #02612** | | | | | Reference Interval | | Units |
| --- | --- | --- | --- | --- | --- | --- | --- | --- |
|  | Day 0 | Day 15 | Day 29 | Day 119 | Day 176 | Low | High |  |
| **Plasma Free Hemoglobin** | 0.40 | 0.30 | 0.30 | 0.40 | 0.30 | *0.00* | *1.04* | *g/L* |

Study Animal 02612 - CBC results.

|  | **Study Animal #02612** | | | | | Reference Interval | | Units |
| --- | --- | --- | --- | --- | --- | --- | --- | --- |
|  | Day 0 | Day 15 | Day 29 | Day 119 | Day 176 | Low | High |  |
| **RBC** | 11.70 | 10.40 | 11.40 | 11.80 | 13.20 | *9.10* | *14.70* | *10^12^/L* |
| **Hemoglobin** | 11.60 | 10.50 | 12.00 | 12.20 | 12.10 | *9.60* | *14.80* | *g/dL* |
| **Hematocrit** | 0.37 | 0.34 | 0.37 | 0.37 | 0.39 | *0.27* | *0.43* | *L/L* |
| **MCV** | 32.00 | 33.00 | 32.00 | 31.00 | 30.00 | *25.00* | *35.00* | *fL* |
| **MCHC** | 31.00 | 31.00 | 32.00 | 33.00 | 31.00 | *31.00* | *39.00* | *g/dL* |
| **MCH** | 10.00 | 10.00 | 11.00 | 10.00 | 9.00 | *9.00* | *12.00* | *pg* |
| **WBC** | 6.80 | 5.40 | 5.90 | 4.40 | 7.40 | *3.40* | *10.80* | *10^9^/L* |
| ***Neutrophil ratio*** | *47.00* | *41.00* | ***12.00*** | *33.00* | ***69.00*** | *21.00* | *64.00* | *%* |
| ***Eosinophil ratio*** | *4.00* | *5.00* | *9.00* | *4.00* | *1.00* | *1.00* | *19.00* | *%* |
| ***Basophil ratio*** | *0.00* | *0.00* | *1.00* | *0.00* | *0.00* | *0.00* | *2.00* | *%* |
| ***Lymphocyte ratio*** | *38.00* | *50.00* | ***73.00*** | *60.00* | ***28.00*** | *30.00* | *71.00* | *%* |
| ***Monocyte ratio*** | ***11.00*** | *4.00* | *5.00* | *3.00* | *2.00* | *1.00* | *9.00* | *%* |
| **Platelets** | 451.00 | **844.00** | 516.00 | 286.00 | 352.00 | *119.00* | *765.00* | *10^9^/L* |
| **Reticulocytes** | 0.12 | **0.48** | 0.23 | 0.17 | 0.26 | *0.03* | *0.36* | *%* |

Study Animal 02612 - Coagulation results.

|  | **Study Animal #02612** | | | | | Reference Interval | | Units |
| --- | --- | --- | --- | --- | --- | --- | --- | --- |
|  | Day 0 | Day 15 | Day 29 | Day 119 | Day 176 | Low | High |  |
| **PR** | **28.00** | **30.00** | **27.00** | **24.00** | **32.00** | 33.00 | 82.00 | % |
| **aPTT** | 36.40 | 27.10 | 32.10 | 36.40 | 29.30 | 22.00 | 44.00 | sec |
| **Fibrinogen** | 2.60 | 2.80 | 1.60 | 1.90 | **1.20** | 1.50 | 4.90 | g/L |

### Study Animal 02680 - Biochemistry results.

|  | **Study Animal #02680** | | | | | Reference Interval | | Units |
| --- | --- | --- | --- | --- | --- | --- | --- | --- |
|  | Day 0 | Day 14 | Day 32 | Day 123 | Day 180 | Low | High |  |
| **Urea** | 5.60 | 6.00 | 5.60 | 6.00 | 8.30 | *2.30* | *10.20* | *mmol/L* |
| **Creatinine** | 87.00 | 79.00 | 99.00 | 87.00 | 87.00 | *68.00* | *150.00* | *µmol/L* |
| **Glucose** | 3.50 | 3.80 | 3.70 | 4.20 | 4.40 | *2.40* | *9.50* | *mmol/L* |
| **Sodium** | 147.00 | 143.00 | 143.00 | 146.00 | 142.00 | *142.00* | *155.50* | *mmol/L* |
| **Potassium** | 4.50 | 4.80 | 4.50 | 4.70 | **3.50** | *3.90* | *6.30* | *mmol/L* |
| **Chloride** | 109.00 | 109.00 | 104.00 | 106.00 | 104.00 | *98.00* | *112.00* | *mmol/L* |
| **Bicarbonate** | 29.00 | 27.40 | 29.50 | 27.80 | 30.70 | *19.00* | *32.00* | *mmol/L* |
| **Plasma Proteins** | 75.60 | 72.70 | 67.70 | 71.00 | 75.70 | *60.00* | *86.00* | *g/L* |
| **Calcium** | 2.23 | 2.41 | 2.35 | 2.26 | 2.47 | *2.02* | *2.81* | *mmol/L* |
| **Phosphorus** | 2.02 | 1.92 | 2.23 | 2.27 | 1.28 | *1.09* | *3.27* | *mmol/L* |
| **Magnesium** | 0.86 | 0.78 | 0.81 | 0.85 | 0.97 | *0.64* | *1.11* | *mmol/L* |
| **Total Bilirubin** | <3 | <3 | <3 | <3 | <3 | *0.00* | *6.00* | *µmol/L* |
| **Alkaline Phosphatase** | 91.00 | 88.00 | 94.00 | 144.00 | 107.00 | *37.00* | *299.00* | *U/L* |
| **Gamma Glutamyl Transferases** | 51.00 | 53.00 | 37.00 | 57.00 | 51.00 | *33.00* | *84.00* | *U/L* |
| **AST** | 119.00 | 79.00 | 78.00 | 137.00 | 123.00 | *67.50* | *253.50* | *U/L* |
| **ALT** | 28.00 | 17.00 | 20.00 | 31.00 | 28.00 | *9.00* | *46.50* | *U/L* |

Study Animal 02680 - Plasma Free Haemoglobin results

|  | **Study Animal #02680** | | | | | Reference Interval | | Units |
| --- | --- | --- | --- | --- | --- | --- | --- | --- |
|  | Day 0 | Day 14 | Day 32 | Day 123 | Day 180 | Low | High |  |
| **Plasma Free Hemoglobin** | 0.50 | 0.40 | 0.30 | 0.50 | 0.20 | *0.00* | *1.04* | *g/L* |

Study Animal 02680 - CBC results

|  | **Study Animal #02680** | | | | | Reference Interval | | Units |
| --- | --- | --- | --- | --- | --- | --- | --- | --- |
|  | Day 0 | Day 14 | Day 32 | Day 123 | Day 180 | Low | High |  |
| **RBC** | 12.30 | 10.90 | 11.90 | 13.40 | 12.90 | *9.10* | *14.70* | *10^12^/L* |
| **Hemoglobin** | 12.60 | 11.00 | 12.20 | 12.90 | 11.90 | *9.60* | *14.80* | *g/dL* |
| **Hematocrit** | 0.36 | 0.32 | 0.36 | 0.38 | 0.36 | *0.27* | *0.43* | *L/L* |
| **MCV** | 29.00 | 29.00 | 30.00 | 28.00 | 28.00 | *25.00* | *35.00* | *fL* |
| **MCHC** | 35.00 | 34.00 | 34.00 | 34.00 | 33.00 | *31.00* | *39.00* | *g/dL* |
| **MCH** | 10.00 | 10.00 | 10.00 | 10.00 | 9.00 | *9.00* | *12.00* | *pg* |
| **WBC** | 9.30 | **12.90** | 8.50 | 7.10 | 6.40 | *3.40* | *10.80* | *10^9^/L* |
| ***Neutrophil ratio*** | *38.00* | *57.00* | *24.00* | *26.00* | *42.00* | *21.00* | *64.00* | *%* |
| ***Eosinophil ratio*** | *6.00* | *2.00* | *3.00* | *2.00* | *1.00* | *1.00* | *19.00* | *%* |
| ***Basophil ratio*** | *1.00* | *0.00* | *1.00* | *1.00* | *0.00* | *0.00* | *2.00* | *%* |
| ***Lymphocyte ratio*** | *46.00* | *38.00* | *70.00* | *68.00* | *44.00* | *30.00* | *71.00* | *%* |
| ***Monocyte ratio*** | *9.00* | *3.00* | *2.00* | *3.00* | ***13.00*** | *1.00* | *9.00* | *%* |
| **Platelets** | 515.00 | 581.00 | 414.00 | 369.00 | 477.00 | *119.00* | *765.00* | *10^9^/L* |
| **Reticulocytes** | 0.20 | 0.27 | 0.24 | 0.07 | 0.15 | *0.03* | *0.36* | *%* |

|  | **Study Animal #02680** | | | | | **Reference Interval** | | **Units** |
| --- | --- | --- | --- | --- | --- | --- | --- | --- |
|  | Day 0 | Day 14 | Day 32 | Day 123 | Day 180 | Low | High |  |
| **PR** | **28.00** | **27.00** | **28.00** | **24.00** | **31.00** | *33.00* | *82.00* | *%* |
| **aPTT** | 30.10 | 29.40 | 27.70 | 29.10 | 27.60 | *22.00* | *44.00* | *sec* |
| **Fibrinogen** | 2.60 | 2.60 | 1.60 | 1.70 | 2.40 | *1.50* | *4.90* | *g/L* |

Study Animal 02680 - Coagulation results

Study Animal 02653 - Biochemistry results

|  | **Study Animal #02653** | | | | | Reference Interval | | Units |
| --- | --- | --- | --- | --- | --- | --- | --- | --- |
|  | Day 0 | Day 14 | Day 32 | Day 123 | Day 180 | Low | High |  |
| **Urea** | 6.00 | 9.10 | 6.10 | 6.90 | 9.40 | *2.30* | *10.20* | *mmol/L* |
| **Creatinine** | 103.00 | 85.00 | 109.00 | 100.00 | 106.00 | *68.00* | *150.00* | *µmol/L* |
| **Glucose** | 3.70 | 4.00 | 3.90 | 4.20 | 4.60 | *2.40* | *9.50* | *mmol/L* |
| **Sodium** | 146.00 | 143.00 | 145.00 | 151.00 | 144.00 | *142.00* | *155.50* | *mmol/L* |
| **Potassium** | 5.00 | 4.80 | 4.40 | 5.50 | **3.50** | *3.90* | *6.30* | *mmol/L* |
| **Chloride** | 109.00 | 108.00 | 107.00 | 111.00 | 98.00 | *98.00* | *112.00* | *mmol/L* |
| **Bicarbonate** | 28.30 | 27.10 | 27.80 | 27.10 | **33.50** | *19.00* | *32.00* | *mmol/L* |
| **Plasma Proteins** | 76.50 | 73.90 | 66.40 | 67.20 | 77.20 | *60.00* | *86.00* | *g/L* |
| **Calcium** | 2.48 | 2.40 | 2.32 | 2.29 | 2.35 | *2.02* | *2.81* | *mmol/L* |
| **Phosphorus** | 1.72 | 2.44 | 2.85 | 2.70 | 2.49 | *1.09* | *3.27* | *mmol/L* |
| **Magnesium** | 0.93 | 0.87 | 0.87 | 1.04 | 0.99 | *0.64* | *1.11* | *mmol/L* |
| **Total Bilirubin** | <3 | <3 | <3 | <3 | <3 | *0.00* | *6.00* | *µmol/L* |
| **Alkaline Phosphatase** | 214.00 | 163.00 | 296.00 | 269.00 | 191.00 | *37.00* | *299.00* | *U/L* |
| **Gamma Glutamyl Transferases** | 68.00 | 71.00 | 59.00 | 79.00 | 83.00 | *33.00* | *84.00* | *U/L* |
| **AST** | 118.00 | 76.00 | 93.00 | 144.00 | 205.00 | *67.50* | *253.50* | *U/L* |
| **ALT** | 24.00 | 15.00 | 22.00 | 23.00 | 24.00 | *9.00* | *46.50* | *U/L* |

Study Animal 02653 - Plasma Free Haemoglobin results

|  | **Study Animal #02653** | | | | | Reference Interval | | Units |
| --- | --- | --- | --- | --- | --- | --- | --- | --- |
|  | Day 0 | Day 14 | Day 32 | Day 123 | Day 180 | Low | High |  |
| **Plasma Free Hemoglobin** | 0.40 | 0.30 | 0.30 | 0.40 | 0.20 | *0.00* | *1.04* | *g/L* |

Study Animal 02653 - CBC results for

|  | **Study Animal #02653** | | | | | Reference Interval | | Units |
| --- | --- | --- | --- | --- | --- | --- | --- | --- |
|  | Day 0 | Day 14 | Day 32 | Day 123 | Day 180 | Low | High |  |
| **RBC** | 13.20 | 11.20 | 11.00 | 12.90 | 14.10 | *9.10* | *14.70* | *10^12^/L* |
| **Hemoglobin** | 13.10 | 11.30 | 11.20 | 12.90 | 13.20 | *9.60* | *14.80* | *g/dL* |
| **Hematocrit** | 0.38 | 0.34 | 0.34 | 0.38 | 0.40 | *0.27* | *0.43* | *L/L* |
| **MCV** | 29.00 | 30.00 | 31.00 | 29.00 | 28.00 | *25.00* | *35.00* | *fL* |
| **MCHC** | 34.00 | 33.00 | 33.00 | 34.00 | 33.00 | *31.00* | *39.00* | *g/dL* |
| **MCH** | 10.00 | 10.00 | 10.00 | 10.00 | 9.00 | *9.00* | *12.00* | *pg* |
| **WBC** | 10.30 | 7.00 | 5.00 | 5.20 | 5.30 | *3.40* | *10.80* | *10^9^/L* |
| ***Neutrophil ratio*** | *44.00* | *31.00* | ***13.00*** | *30.00* | *55.00* | *21.00* | *64.00* | *%* |
| ***Eosinophil ratio*** | ***25.00*** | *13.00* | *7.00* | *8.00* | *3.00* | *1.00* | *19.00* | *%* |
| ***Basophil ratio*** | *0.00* | *2.00* | *2.00* | *1.00* | *0.00* | *0.00* | *2.00* | *%* |
| ***Lymphocyte ratio*** | *30.00* | *53.00* | ***74.00*** | *58.00* | *38.00* | *30.00* | *71.00* | *%* |
| ***Monocyte ratio*** | *1.00* | *1.00* | *4.00* | *3.00* | *4.00* | *1.00* | *9.00* | *%* |
| **Platelets** | 353.00 | 703.00 | 246.00 | 400.00 | 344.00 | *119.00* | *765.00* | *10^9^/L* |
| **Reticulocytes** | 0.29 | 0.36 | 0.29 | 0.10 | 0.17 | *0.03* | *0.36* | *%* |

Study Animal 02653 - Coagulation results

|  | **Study Animal #02653** | | | | | Reference Interval | | Units |
| --- | --- | --- | --- | --- | --- | --- | --- | --- |
|  | Day 0 | Day 14 | Day 32 | Day 123 | Day 180 | Low | High |  |
| **PR** | **31.00** | **31.00** | **30.00** | **30.00** | 35.00 | *33.00* | *82.00* | *%* |
| **aPTT** | 36.30 | 30.00 | 34.20 | 29.60 | 32.60 | *22.00* | *44.00* | *sec* |
| **Fibrinogen** | 2.30 | 3.10 | 1.50 | 1.70 | 2.60 | *1.50* | *4.90* | *g/L* |

***Table S6:*** *AST: Aspartate Aminotransferase; ALT: Alanine Aminotransferase; RBC: Red Blood Cell; MVC: Mean Corpuscular Volume; MCHC: Mean Corpuscular Hemoglobin Concentration; MCH: Mean Corpuscular Hemoglobin; WBC: White Blood count; PR: Prothrombin Time Ratio; aPTT: Activated Partial Thromboplastin Time*


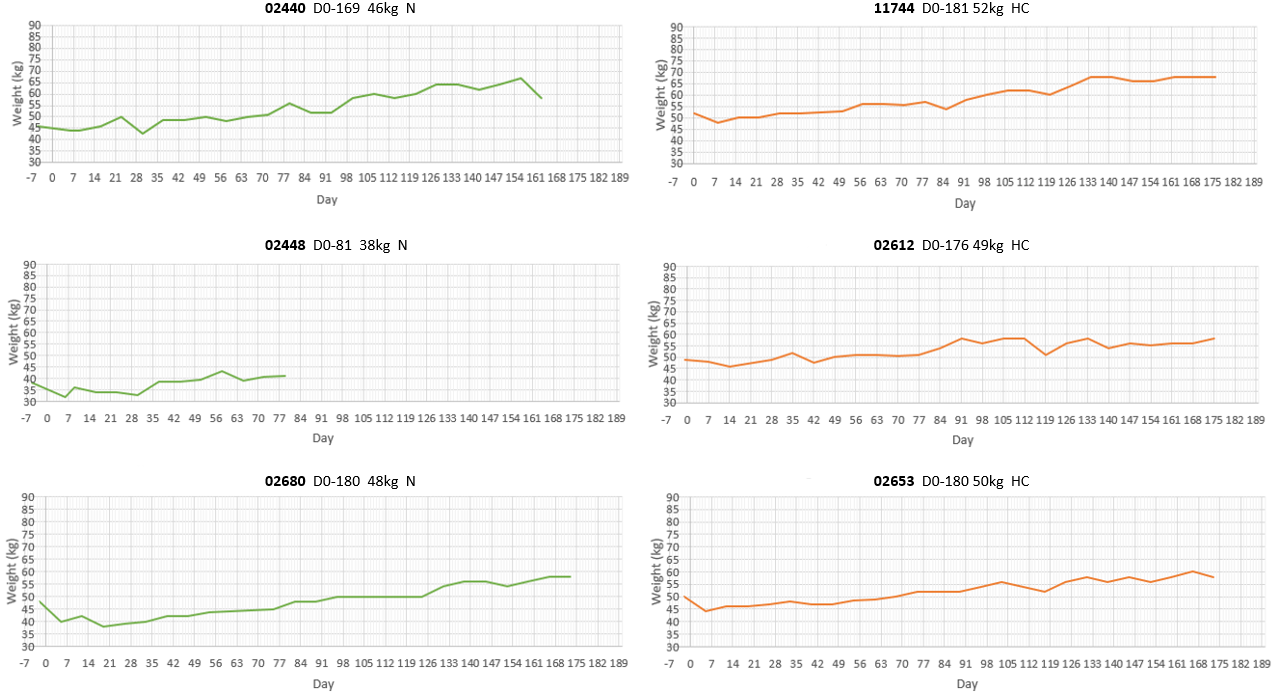
**Figure S7**

*Weight overtime for each study animal. The legends for each study case shows study animal, termination day, weight at surgery and N (normal) or HC (heparin-coated) sub-groups.*
